# Roles of exosomes in immunotherapy for solid cancers

**DOI:** 10.1038/s41419-024-06494-z

**Published:** 2024-02-01

**Authors:** Cong Lyu, Haifeng Sun, Zhenqiang Sun, Yang Liu, Qiming Wang

**Affiliations:** 1 Department of Internal Medicine, The Affiliated Cancer Hospital of Zhengzhou University & Henan Cancer Hospital, Zhengzhou, 450008 China; 2 Department of Molecular Pathology, The Affiliated Cancer Hospital of Zhengzhou University & Henan Cancer Hospital, Zhengzhou, 450008 China; 3 Department of Colorectal Surgery, The First Affiliated Hospital of Zhengzhou University, Zhengzhou, 450052 China; 4 Department of Radiotherapy, The Affiliated Cancer Hospital of Zhengzhou University & Henan Cancer Hospital, Zhengzhou, 450008 China

**Keywords:** Cancer microenvironment, Cancer immunotherapy, Nanoparticles

## Abstract

Although immunotherapy has made breakthrough progress, its efficacy in solid tumours remains unsatisfactory. Exosomes are the main type of extracellular vesicles that can deliver various intracellular molecules to adjacent or distant cells and organs, mediating various biological functions. Studies have found that exosomes can both activate the immune system and inhibit the immune system. The antigen and major histocompatibility complex (MHC) carried in exosomes make it possible to develop them as anticancer vaccines. Exosomes derived from blood, urine, saliva and cerebrospinal fluid can be used as ideal biomarkers in cancer diagnosis and prognosis. In recent years, exosome-based therapy has made great progress in the fields of drug transportation and immunotherapy. Here, we review the composition and sources of exosomes in the solid cancer immune microenvironment and further elaborate on the potential mechanisms and pathways by which exosomes influence immunotherapy for solid cancers. Moreover, we summarize the potential clinical application prospects of engineered exosomes and exosome vaccines in immunotherapy for solid cancers. Eventually, these findings may open up avenues for determining the potential of exosomes for diagnosis, treatment, and prognosis in solid cancer immunotherapy.

## Facts

- Cancer immunotherapy has greatly promoted the development of cancer treatment. However, due to the influence of tumour immune microenvironment, the response of solid cancers to immunotherapy is limited.
- The biological significance of exosomes has been deeply studied and has greatly developed in cancer diagnosis and treatment.
- Exosomes serve as diagnostic biomarkers, therapeutic targets, and drug delivery platforms and can be used for vaccine development.
- Although exosome-based strategies have yielded benefits for improving the effects of anticancer immunotherapy, huge barriers remain in their clinical application for solid cancers.

## Open questions

- Mechanisms and pathways that exosomes affect immunotherapy for solid cancers?
- How do exosomes derived from different cell types affect immunotherapy for solid cancers?
- The application of engineering exosomes and exosomes vaccines in immunotherapy of solid tumours.
- How to dissolve problems faced by the clinical transformation of exosomes in immunotherapy for solid cancers?

## Introduction

Cancer immunotherapy has greatly promoted the development of cancer treatment and improved the survival rate of patients with advanced malignant tumours [1]. However, the response of solid tumours to immunotherapy is limited. In contrast, the differential expression of unique immunoregulatory molecules significantly promotes the interaction between haematological tumour cells and immune cells, making the response rate of haematologic malignancies to immunotherapy significantly higher than that of solid tumours [2, 3]. Research progress on the efficacy of immunotherapy for solid tumours has been slow, mainly due to the following factors: first, the effects of hypoxic tumour immunosuppressive microenvironment on T-cell proliferation, differentiation, and effector activity; second, the effects of extracellular matrix, tumour blood vessels, and cancer-related fibroblasts on the penetration of therapeutic agents, T-cell proliferation, T-cell cytotoxicity, and T-cell immune infiltration; and third, the effects of regulatory T cells (Tregs), myeloid-derived suppressor cells (MDSCs), and tumour-associated macrophages (TAMs) on immunosuppressive effects in the tumour microenvironment (TME) [4–7].

Tumours refer to not only different subsets of tumour cells but also cancer-associated fibroblasts (CAFs), innate and adaptive immune cells (such as monocytes/macrophages, dendritic cells, neutrophils, MDSCs, natural killer cells (NKs), T cells, and B cells), blood vessel systems, secreted factors, and a wide range of extracellular matrix (ECM) networks, collectively known as the TME [8, 9]. Acidity, hypoxia, and nutritional deficiency are common characteristics of the TME, providing strong selection pressure during tumour progression. Cancer develops in a harsh and complex TME, and intercellular communication occurs through various signalling networks, which play a key role in tumour progression and metastasis [10, 11]. The intercellular communication mediators in the TME include cytokines, chemokines, growth factors, signalling molecules and their receptors, tumour cells, interstitial cells, and extracellular vesicles.

The signal transduction function of exosomes plays an important role in tumour cells and the TME. Exosomes are membranous vesicles with a diameter of ~30–150 nm that are released outside the cell after the fusion of intracellular multivesicular bodies with the cell membrane, which distinguishes them from microvesicles (100–1000 nm) and apoptotic bodies (400–4000 nm) [12–14]. Exosomes are secreted by body fluids and a variety of cells in the human body, including immune cells, endothelial cells, smooth muscle cells, platelets, etc. As a “bridge” for material transmission and mediating intercellular communication, exosomes contain proteins, lipids, nucleic acids, metabolites and other substances obtained from the host cell [15–18]. Exosomes have a typical phospholipid bilayer membrane structure and low immunogenicity and can more efficiently cross the blood-brain barrier and placental barrier. Exosomes transmit biological information such as proteins and genetic materials from host cells to recipient cells, change their physiological and pathological status, or activate the next step of signalling pathways, possessing great potential as natural drug carriers [19, 20]. As the main carriers of intercellular signal transmission, exosomes play a crucial role in the complex communication network between tumour cells and immune cells. On the one hand, exosomes help immune cells communicate with each other, thereby activating downstream effector cells. On the other hand, they present tumour cell-specific antigens to the immune system, inhibiting the immune escape of tumour cells [21, 22].

In this review, we summarize the composition and sources of exosomes in the solid cancer immune microenvironment and the role of exosomes in immunotherapy for solid cancers. We further elaborate on the potential mechanisms and pathways by which exosomes influence immunotherapy for solid cancers. In addition, we describe the potential clinical application prospects of engineered exosomes and exosome vaccines in immunotherapy for solid cancers.

## The biogenesis and biological functions of exosomes

Exosomes were first reported by Wolf in 1967 [23]. Several years later, Johnstone et al. [24] first observed exosomes during the maturation of sheep reticulocytes in vitro and studied their relationship with plasma membrane activity in the 1980s. They demonstrated that each vesicle formed during the maturation of the membrane had multiple plasma membrane functions [25], and the release of exosomes was the main pathway for the externalization of obsolete membrane proteins [26]. In 1996, Raposo et al. [27] also observed exosomes in B lymphocytes, which showed that they contained MHC class II molecules and had essential biological activities such as stimulating T cell proliferation and inhibiting tumour growth. In 2014, studies reported the detection of exosomes in different types of cells cultured in vitro, such as dendritic cells (DCs), epithelial cells, tumour cells, and mesenchymal stem cells (MSCs) [28].

When exosomes were first discovered, they were believed to be “garbage bags” used by cells to eliminate unnecessary proteins and excrete metabolic waste within cells, but they did not receive widespread attention. With the advancement of research technology, more and more studies have found that exosome, as a bridge for communication between cells, can participate in various signal transduction between cells. Meanwhile, by transporting different types of biomolecules, exosomes can also achieve component exchange between cells, mediating various physiological and pathological processes of cells.

## The biogenesis of exosomes

Research has shown that exosomes are formed from donor cells which are activated under different factors such as inflammation, hypoxia, oxidative stress, aging, and cell apoptosis [29]. The formation of exosomes mainly involves processes such as endocytosis, cargo sorting, and transportation, and involves various protein molecules [30]. Firstly, the endosomal sorting complex required for transport-0 (ESCRT-0) binds to specific receptors on the extracellular surfaces of early endosome through ubiquitination binding sites, inward budding in a reverse manner, and selectively encapsulate part of the cytoplasm to form intraluminal vesicles (ILVs), which form budding vesicles under the action of ESCRT-I and II, and then separate from the endosomal membrane under the scission action of ESCRT-III, forming mature late endosomes, namely multivesicular bodies (MVBs). Based on different biochemical characteristics, some MVBs are transported to lysosomes for proteomic degradation, while others are transported to the cell membrane and fused with them, releasing their contents into the extracellular microenvironment. These ILVs released into the extracellular environment are called exosomes. The secretion of exosomes is regulated by various molecules, especially Rabs and Ral proteins, such as Rab5, Rab27, Rab35, RalA, and RalB proteins [31, 32]. Studies have shown that knocking down Rab27a or Rab27b in HeLa cells reduces the docking of MVEs with the plasma membrane [33]. Partial secretion of exosomes from tumours in vivo can promote tumour progression. Studies have shown that Rab27a can effectively inhibit the secretion of exosomes from tumour cells, thereby interfering with tumour progression [34]. Research has shown that Rab27-dependent exosomes can inhibit chronic inflammation and produce acute responses to inflammatory stimuli [35–37].

## The biological functions of exosomes

Exosomes have a wide range of sources and distributions, diverse functions, and are closely related to their respective source cells. Different exosomes have different biological characteristics and can produce different effects due to factors such as their contents, tissue microenvironment, and receptor cells. There are three main ways in which exosomes exert their effects [12]: The first type is by binding ligands on the exosome membrane to receptors on the target cell membrane to transmit intercellular information; The second type is the fusion of exosomes and target cells to release various proinflammatory factors and nucleic acids, which facilitate information transmission; The third type is that exosomes release intracellular substances that act on the surface receptors of target cells, completing information transmission and producing biological effects. Through the above pathways, exosomes transport their biological information to surrounding target cells or through bodily fluids such as blood, which is then taken up by distant tissue cells, thereby affecting the basic function and gene expression of the target cells. Almost all cells can produce exosomes spontaneously or under certain stimulation conditions. The exosomes produced by different cells have different functions and are involved in a series of physiological and pathological processes, such as immune regulation, antigen presentation, tissue repair, cancer occurrence and development, etc.

Exosomes have immune-regulatory functions. Exosomes carry various immune-related molecules, which regulate the immune response. TNF family proteins such as Fas ligands (FasL), tumour necrosis factor-related apoptosis-inducing ligands (TRAIL), and CD40 ligands can be carried on exosomes derived from source cells, which can induce target cell apoptosis. For example, there are FasL and TRAIL expressed in placental tissue on the surface of exosomes. These two signalling molecules trigger T-cell apoptosis through contact with target cells, thereby maintaining normal pregnancy [38]. Exosomes generally complete antigen presentation, immune activation inhibition, and immune tolerance processes by transmitting multiple biological molecules. Among them, the exosomes secreted by antigen-presenting cells can carry and present related complexes, achieving the goal of immunotherapy by weakening or enhancing the immune response [39]. In addition, exosomes also carry many immune-related molecules, including lysosomal-associated membrane proteins-1 and-2 (LAMP-1 and LAMP-2) [40], immunoglobulin superfamily member 8 (IGSF8), and MHCs, which can specifically bind to related peptide chains and induce immune responses. Exosomes can present various complexes, enhance or weaken the body’s immune response, and can be used to regulate excessive immunity (autoimmune diseases) and insufficient immunity (immunodeficiency disorders). Studies have shown that the content and activity of exosomes in the airway of asthma patients are different from those in healthy individuals. Myeloid suppressive cells transfer mitochondria to T cells through exosomes, indicating that exosomes may mediate crucial immune regulatory effects [41]. Exosomes also have the functions of inducing cell division and differentiation, which can foster the differentiation of MSCs into different cells to replace damaged tissue cells, and are applied in the field of regenerative medicine. The exosomes derived from MSCs play a pivotal role in osteochondral regeneration. Studies have shown that exosomes secreted by MSCs derived from human Wharton’s jelly can promote the migration, proliferation, and chondrocyte proliferation of bone marrow mesenchymal stem cells [42]. Exosomes, as decisive carriers of intercellular communication, directly participate in the occurrence and development of tumours. Fibroblasts activated by exosomes can differentiate into CAFs, which can lead to excessive collagen deposition and ECM remodelling, promoting the formation of the tumour microenvironment [43]. The growth and metastasis of tumour cells require blood vessels to supplement oxygen and nutrients, and exosomes are also involved in the regulation of pathological angiogenesis [44]. Under hypoxic conditions, the content of miR-23a in exosomes derived from lung cancer cells significantly increased. At the same time, miR-23a was transported to endothelial cells and directly inhibited the expression of target genes proline hydroxylase 1 and proline hydroxylase 2 (PhD1 and PhD2), causing hypoxia-inducible factor-1 α (HIF-1α) accumulation within endothelial cells promotes angiogenesis and tumour invasion and metastasis [45].

## Exosomes derived from different sources

Exosomes of different cell origins have distinct functions. Immune cells are key components of the TME. Exosomes are a double-edged sword in the TME. Tumour cells can regulate immune cells to promote tumour growth. In contrast, under special circumstances, immune cells can activate the immune response in the TME through exosomes to inhibit the growth of tumour cells [46]. An increasing number of studies have shown that most types of immune cells, such as B cells, T cells, natural killer cells, dendritic cells, and macrophages, secrete exosomes [47]. The exosomes produced by immune cells contain proteins, nucleic acids, and specific antigens from donor cells. Surface antigens of exosomes have the ability to directly stimulate receptor cells and can target specific types of receptor cells to play a regulatory role. The sealed lipid bilayer on the surface of exosomes can effectively protect molecular transporters and stably transfer various molecules from donor cells to recipient cells [30]. The combination of these characteristics endows exosomes with a powerful function in influencing the host immune response. Exosomes secreted from tumour cells are called tumour-derived exosomes (TDEs), which have more specificity than the general characteristics of exosomes [48]. A large variety of evidence shows that exosomes secreted by host cells or cancer cells are involved in the genesis, growth, invasion and metastasis of tumours. Communication between immune cells and cancer cells through exosomes plays a dual role in regulating tumours [49, 50].

## Immune cells-derived exosomes

### NKs-derived exosomes (NK-Exo)

**Fig. 1:**
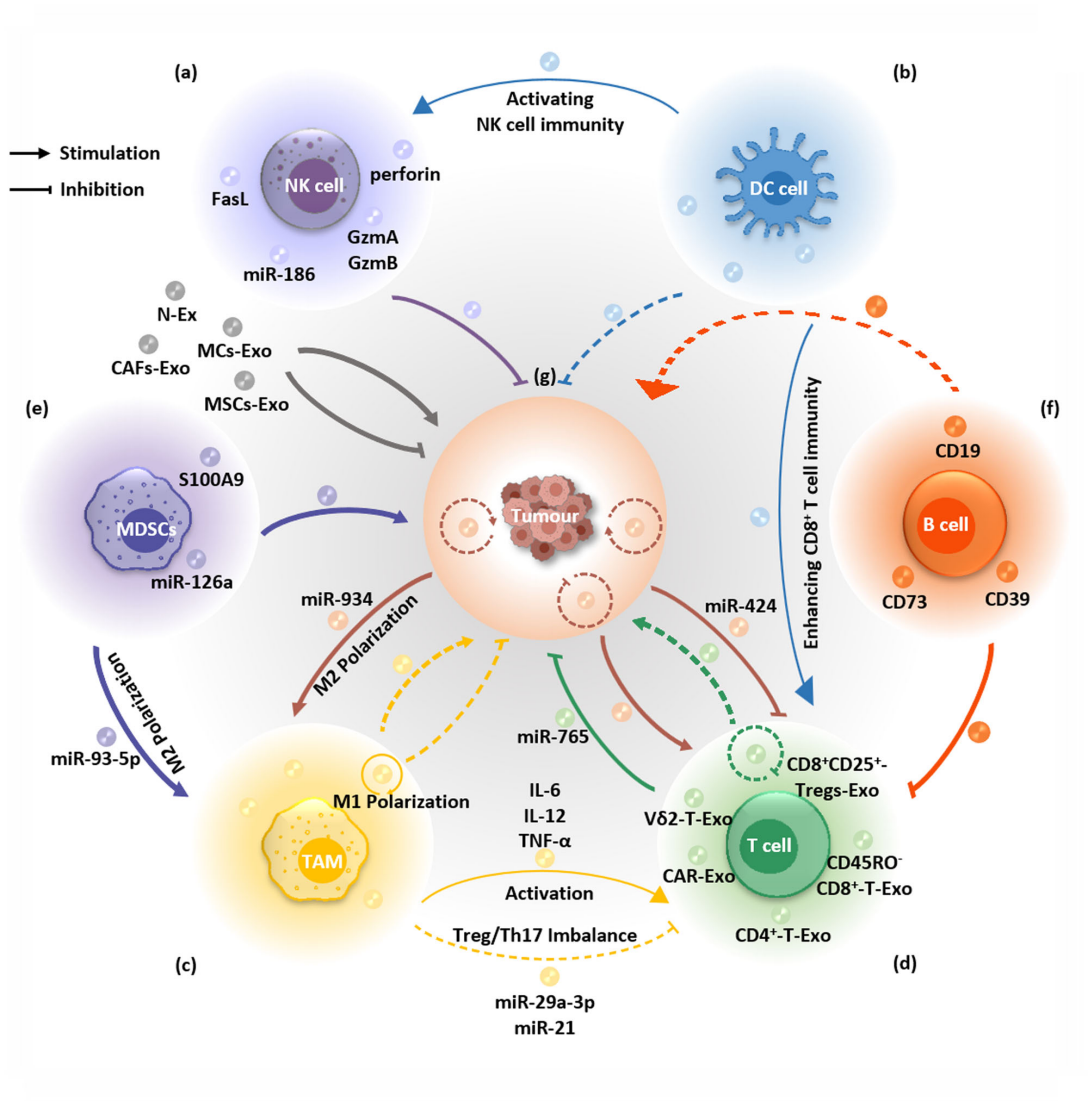
Exosomes derived from different sources. **a** NK cells secrete exosomes containing typical NK markers, cytotoxic proteins and miR-186, which mediate cytotoxicity against solid tumours. **b** Dex suppress tumour progression by activating the antitumour immunity of NK cells and enhancing CD8^+^ T-cell-mediated anticancer immunity. **c** TAMs secrete exosomes containing cytotoxic proteins that mediate cytotoxicity against solid tumours and miRNAs that promote solid tumour progression. **d** Vδ2 T-cell-derived exosomes, CAR T-cell-derived exosomes, CD4^+^-T-Exo, CD45RO^-^CD8^+^-T-Exo and exosomal miR-765 suppress solid tumour growth. While CD8^+^CD25^+^-Tregs-Exo exhibit immunosuppressive effects by inhibiting CTL antitumour immune responses. **e** The secretion of G-MDSC-derived exosomal miR-93-5p promoted the differentiation of M-MDSC into M2 macrophages, thereby promoting solid tumour progression. **f** CD19-positive exosomes released by B cells contain high levels of CD39 and CD73 molecules, which weaken the antitumour effects through inhibiting the activation of CD8^+^ T cells. **g** Tumour-derived exosomes exert both antitumour and tumour-promoting functions.

NKs are significant components of innate immunosurveillance of tumours and the TME [51]. NK cells have attracted widespread attention in recent years. They not only directly kill tumour cells but also rapidly express a variety of cytokines and chemokines, recruit other immune cells and promote the adaptive immune response of T cells and B cells. Moreover, NK cells were activated by IgG antibodies to produce antibody-dependent cell-mediated cytotoxicity (ADCC). In addition, it rarely triggers an autoimmune response but instead promotes immune balance and combats autoimmune diseases [52]. The multiple beneficial characteristics of NK cells make them ideal targets for immunotherapy. NK cells secreted exosomes containing typical NK markers and cytotoxic proteins (e.g., perforin molecules, Fas ligand, granzyme A & B (GzmA & GzmB), and granulysin), which mediated cytotoxicity against different solid tumours (e.g., breast cancer and neuroblastoma), resulting in cytotoxicity of cancer cells [53–55] (Fig. 1a). Studies detected diminished granzyme A substrate SET and HMG2 proteins and release of cytochrome C from mitochondria in neuroblastoma cells treated with NK cell-derived exosomes, which indicated caspase-independent and caspase-dependent cell death pathways mediated by NK cell-derived exosomes, respectively [55]. Studies not only demonstrated that exosomes derived from NK-92MI cells (NK-92 Exo) inhibited the proliferation and induced the apoptosis of melanoma cells in vitro but also proved that NK-92 Exo could inhibit tumour growth in melanoma xenograft mice in vivo [56]. Some cytokines, such as interleukin-15 (IL-15), could enhance the antitumour ability of NK cell-derived exosomes against glioblastoma, breast cancer and thyroid cancer [57]. In addition, NK cell-derived exosomes could also carry tumour suppressor microRNAs (miRNAs), which prevent neuroblastoma growth and TGFβ1-dependent immune escape [58]. Studies evaluated the cytotoxic effect of exosomes derived from expanded natural killer cells (eNK-EXO) against ovarian cancer cells by itself and by exosomes loaded with cisplatin [59]. Transcription analysis further revealed that eNK-EXO could activate the antitumour immune function of NK cells [59].

### Dendritic cells-derived exosomes (Dex)

As the main antigen-presenting cells, DCs have powerful functions in the uptake, processing, and presentation of antigens and are one of the main “messengers” that transmit cancer cell signals to T cells [60]. Studies have reported that DCs complete the entire process of antitumour immunity by forming exosomes [61]. Exosomes derived from α-fetoprotein (AFP)-expressing DCs could suppress tumour progression in mice with ectopic, orthotopic and carcinogen-induced HCC tumours by remodelling the tumour immune microenvironment [62]. Studies revealed that DC-derived exosomes (Dex) loaded with patient-specific neoantigens could markedly suppress tumour progression, extend survival, and prohibit lung metastasis in B16F10 melanoma and MC-38 mouse models [63]. Studies have demonstrated that cancer-specific transcription-induced chimeric RNA-loaded DC-derived exosomes enhance CD8^+^ T-cell-mediated anticancer immunity and suppress tumour progression in esophageal cancer [64]. In addition, DC-derived exosomes have been applied in a phase II clinical trial and have been demonstrated to improve the progression-free survival of patients with advanced non-small cell lung cancer (NSCLC) by activating the antitumour immunity of NK cells [65] (Fig. 1b).

### TAMs-derived exosomes

TAMs are the main participants and mediators that promote tumour progression in the TME [66]. Typically, macrophages are polarized into M1-type macrophages or M2-type macrophages [67]. M1-polarized macrophages are activated by cytokines such as IFN-γ and produce proinflammatory and immunostimulant cytokines such as IL12 and TNF-α. However, TAMs are thought to be closer to M2-polarized macrophages, which are activated by Th2 cytokines (IL4, IL10, and IL13) [68, 69]. With the progression of tumours, TAMs can promote the proliferation, invasion, and metastasis of tumour cells, stimulate tumour-related angiogenesis, and inhibit the antitumour immune response [69, 70]. As the relationship between TAMs and tumours is gradually clarified, TAMs are now considered a potential therapeutic target for tumours. Macrophages can secrete exosomes, which participate in communication between different cell types in tumours [71]. Studies on epithelial ovarian cancer (EOC) showed that TAM-derived exosome miRNA chip analysis revealed high expression of miR-29a-3p and miR-21-5p. When the two types of miRNA mimics were transfected into CD4^+^ T cells, STAT3 expression was inhibited, and Treg/T helper 17 (Th17) imbalance was caused. The results showed that TAM-derived exosomes regulated the interaction between TAMs and T cells, resulting in an immunosuppressor microenvironment that promoted the progression and metastasis of EOC tumour cells [72] (Fig. 1c). Although TAMs are largely immunosuppressive, their exosomes have the potential to stimulate rather than restrict antitumour immunity. Studies utilized proteomic techniques to analyse the exosomes secreted by macrophages in mouse colon tumours and their effects on cancer cells and T cells [73]. They found that compared to TAMs, TAM-derived exosomes were associated with molecular signatures associated with Th1/M1 polarization, enhanced inflammation, enhanced immune responses, and more favourable patient outcomes. TAM-derived exosomes also contain bioactive lipids and synthetases, which may alter the proinflammatory signalling of cancer cells [73]. Another study reported that proinflammatory M1 macrophage-derived exosomes have the corresponding functions of M1 macrophages [74]. Exosomes secreted by proinflammatory M1 macrophages can activate the NF-κB pathway in M0 macrophages, which promotes the polarization of M0 macrophages to the M1 phenotype and the release of a large number of inflammatory cytokines (IL-6, IL-12, and TNF-α) to establish a local inflammatory microenvironment. They applied exosomes derived from M1 macrophages to a mouse breast tumour model and discovered that exosomes could induce tumour cell apoptosis and inhibit tumour growth [74] (Fig. 1c).

### T cells-derived exosomes

T cells are major contributors to antitumour immunity and are the most common tumour-infiltrating lymphocytes in the TME. Researchers have found that some T cell-derived exosomes activate the immune activity of immune cells [75]. Exosomes derived from phosphoantigen-expanded Vδ2-T cells could cause apoptosis of Epstein-Barr virus (EBV)-associated gastric carcinoma cells and suppress the growth of EBV-associated gastric carcinoma in Rag2^−/−^ γc^−/−^ mice [76]. CD45RO^−^CD8^+^ T cell-derived exosomes expressing miR-765 were reported to partly inhibit oestrogen-driven endometrial cancer progression by regulating epithelial-mesenchymal transition (EMT) [77] (Fig. 1d). As one of the most important immune cells in the human immune system, CD4^+^ T cells can activate CD8^+^ T cells to differentiate into cytotoxic T lymphocytes (CTLs) through various mechanisms, meanwhile maintaining and strengthening the antitumour response of CTLs. Studies have shown that miR-25-3p, miR-155-5p, miR-215-5p, and miR-375 in exosomes derived from CD4^+^ T cells induced CD8^+^ T cell-mediated antitumour responses in melanoma, which were enhanced under IL-2 stimulation [78] (Fig. 1d). Tregs are an immunosuppressive subgroup of CD4^+^ T cells, playing crucial roles in maintaining self-tolerance and immune homoeostasis. In tumour immunity, Tregs can impair the immune monitoring of cancer in healthy individuals and inhibit the antitumour immune response of tumour-bearing hosts, leading to the progression of various types of cancer [79]. Researchers used a B16F10 mouse melanoma model to demonstrate that activated CD8^+^CD25^+^-Tregs can release exosomes, which expressed molecules related to lymphocyte function (IL-10 and TGF-β) to inhibit CTL antitumour immune responses, demonstrating that CD8^+^CD25^+^-Tregs-derived exosomes have significant immunosuppressive effects [80] (Fig. 1d). Immunotherapy, especially immune checkpoint inhibitors (ICIs) therapy (e.g., anti-programmed cell death protein 1 (PD-1)/programmed cell death ligand 1 (PD-L1) therapy) and adoptive cell therapy (ACT), has made significant clinical progress in cancer treatment. Among them, T cells are crucial for the efficacy of current cancer immunotherapy [75]. Chimeric antigen receptor T cells (CAR-T cells) are T cells that express chimeric antigen receptors across membranes through gene editing and have specific targeting. CAR-T, as a new immunotherapy, has made significant breakthroughs in solid tumour treatment [81]. Recently, CAR-T cells were reported to secrete CAR-containing exosomes (CAR-Exo) that exhibited antitumour effects against some solid tumours, such as breast cancers, lung cancers and ovarian cancers [82] (Fig. 1d).

### MDSCs-derived exosomes

MDSCs are immature myeloid cells that play crucial roles in pathogenic and inflammatory immune responses. MDSCs have powerful immunosuppressive functions, which can inhibit corresponding cellular responses, limit the occurrence and development of inflammation, and promote wound healing. However, tumour cells often alter the generation of bone marrow, affecting the differentiation process of progenitor cells, ultimately leading to the accumulation of MDSCs and even reducing the efficacy of antitumour immunotherapy. At present, there are plenty of reports proving the immunosuppressive effect of MDSCs in TME and its association with poor immune response in antitumour response [83]. Researchers have found that DOX treatment in breast tumour-bearing mice led to the release of IL-13R^+^miR-126a^+^ MDSCs (DOX-MDSCs). DOX-MDSCs-derived miR-126a^+^ exosomes promoted breast tumour lung metastasis by inducing the production of IL-13^+^ Th2 cells and enhancing tumour angiogenesis [84] (Fig. 1e). Studies have demonstrated that MDSCs promoted colorectal cancer cell stemness and tumour growth by releasing exosomes expressing S100A9 [85] (Fig. 1e). The secretion of G-MDSC-derived exosomal miR-93-5p driven by IL-6 promoted the differentiation of M-MDSC into M2 macrophages by downregulating the activity of STAT3 in M-MDSC, thereby promoting colitis-to-cancer transition [86] (Fig. 1e).

### B cells-derived exosomes

B cells are cells with antibody-secreting ability, derived from multifunctional stem cells in the bone marrow. On the one hand, B cells stimulated by antigens will proliferate and differentiate into a large number of plasma cells, secrete antibodies, and circulate in the blood, indirectly exercising their functions through antibody-dependent cell-mediated cytotoxicity (ADCC); On the other hand, as antigen-presenting cells, B cells can directly activate T cells and macrophages, which is particularly prominent in the tumour microenvironment [87]. At present, with the deepening of research on exosomes, the function of B-cell-derived exosomes is gradually being discovered. Researchers have found that CD19-positive exosomes released by B cells contained high levels of CD39 and CD73 molecules, which hydrolyse ATP released by chemotherapy-induced apoptotic tumour cells into adenosine, inhibiting the activation of CD8^+^ T cells during chemotherapy, thereby weakening the antitumour effects of chemotherapy [88] (Fig. 1f). Compared with normal mice, B cells in tumour mice were able to release more levels of exosomes. Compared with healthy individuals, higher levels of CD19-positive exosomes were detected in the serum of tumour patients at different stages. Patients with lower levels of CD19-positive exosomes showed better chemotherapy efficacy. Further research suggested that tumour microenvironment can upregulate the protein levels of HIF-1α in B cells. HIF-1α directly bound to the promoter of Rab27a and promoted the expression of Rab27a protein, ultimately promoting the secretion of more exosomes by B cells. These findings suggested the significance of CD19-positive exosomes in serum for tumour diagnosis and evaluation of chemotherapy efficacy [88] (Fig. 1f).

### Mast cells-derived exosomes

Mast cells (MCs) are myeloid cells present in the connective tissue of the body, containing coarse particles with inflammatory mediators such as histamine. Mast cells have always been believed to be associated with the pathogenesis of allergic and autoimmune diseases. However, it is now recognized that mast cells have decisive impacts on tumour cells and the tumour microenvironment. They are key coordinators of antitumour immunity and regulators of tumour matrix. However, their roles are still controversial, as MCs can exert pro-tumour or antitumour functions in different types of tumours, depending on their location within or around the tumour and their interactions with other components of the tumour microenvironment. Therefore, mast cells are an underrecognized but highly promising target for cancer immunotherapy [89, 90]. Studies have shown that exosomes derived from MCs (MCs-Exo) can transport oncogenic proteins to tumour cells and promote tumour progression through activating signal transduction by ligand-receptor interactions [91]. Researchers isolated exosomes from human mast cell line HMC-1 and detected uptake of exosomes by lung epithelial cell line A549. Subsequently, researchers demonstrated that the exosomes derived from mast cell HMC-1 contain KIT protein, rather than *c-KIT* mRNA, which can activate KIT-SCF signalling transduction, thereby increasing the expression of cyclin D1 and promoting the proliferation of A549 [91] (Fig. 1).

### Neutrophils-derived exosomes (N-Ex)

Neutrophils are acute inflammatory cells that respond to infections. Due to their inability to continue proliferation after maturation and short half-life (6–8 h), it was believed that they can only function as specific immune cells, and therefore have not received much attention. Previous studies have shown that tumour-associated neutrophils can promote tumour occurrence and growth, angiogenesis, extravasation, and metastasis of tumour cells, and inhibit the adaptive immune system, protecting tumour cells from T cell killing [92]. Recent studies revealed that there were multiple types of neutrophils, and their roles in cancer were complex. According to their specific surface markers, neutrophils exhibit heterogeneity and may play different roles in cancer treatment. Through appropriate and effective mediation, neutrophils may become a relevant agent for the body’s antitumour immunity and enhance the efficacy of current immunotherapy treatments [93, 94]. Researchers found that neutrophils in tumour patients who received chemotherapy exhibited more pronounced senescent and proved that exosomes secreted by senescent neutrophils significantly enhanced the drug resistance of breast cancer cells through in vivo and in vitro experiments [95]. Subsequent experiments confirmed that a PIWI-interacting RNA (piRNA-17560) carried by exosomes played key roles and mainly caused ZEB1-mediated EMT in breast cancer cells. Mechanism studies showed that exosomal piRNA-17560 enhanced its stability by binding to 3′UTR of FTO mRNA in breast cancer cells, thereby promoting the expression of FTO. Upregulated FTO promoted the expression of ZEB1 in tumour cells by mediating RNA m^6^A demethylation. Meanwhile, YTHDF2 (an m^6^A reader protein) played a synergistic role. In addition, exosomal piRNA-17560 is produced by stimulating the STAT3 signal of senescent neutrophils by drugs [95]. Another research group isolated exosomes from peripheral blood neutrophils of healthy donors and demonstrated that N-Ex exerted cytotoxicity on tumour cells by activating the apoptotic signalling pathways. In addition, the research group also manufactured exosome-like nanovesicles from neutrophils (NNVs) as new delivery carriers for chemotherapy drugs, which almost completely eliminated tumour growth in gastric cancer xenograft models [96] (Fig. 1).

## CAFs-derived exosomes (CAFs-Exo)

CAFs are the main stromal cells in tumour microenvironment, which can express characteristic biological markers. Studies have shown that CAFs inhibited apoptosis and promoted biological behaviours such as tumour proliferation, invasion, migration, immune escape, and drug resistance by remodelling the extracellular matrix, regulating metabolism, inhibiting immune responses, promoting angiogenesis, and maintaining tumour cell stemness. Therefore, CAFs can serve as bridges for the interaction between tumours and tumour microenvironment. Targeting CAFs to inhibit or even block the occurrence and development of tumours may provide new ideas for the clinical treatment of tumours [97]. Researchers found that miR-500a-5p was simultaneously highly expressed in CAFs-derived exosomes and human breast cancer cells treated with CAFs-derived exosomes, indicating that miR-500a-5p was transferred from CAFs to cancer cells. The author also demonstrated that miR-500a-5p promoted proliferation and metastasis through binding to ubiquitin-specific peptidase 28 (USP28) [98]. Exosomes derived from CAFs carried autophagy-related protein GPR64, which were absorbed by breast cancer cells via tumour microenvironment and activated non-canonical NF-κB signalling pathway, further upregulating the expression of MMP9 and IL-8 and enhancing the invasion and metastasis of cancer cells [99]. Another study on hypoxic breast cancer showed that circHIF1A was upregulated in the exosomes derived from hypoxic CAFs compared with the exosomes derived from normoxic CAFs. It was also proved that circHIF1A was transferred to breast cancer cells by hypoxia CAFs exosomes and promoted sponging miR-580-5p through the expression of CD44, enhancing the formation of breast cancer cell stemness and tumorigenesis [100]. In colorectal cancer (CRC), exosomal miR-92a-3p derived from CAFs activated the Wnt/β-catenin pathway and inhibited mitochondrial apoptosis by attenuating the expression of FBXW7 and MOAP1, thereby promoting cell stemness, EMT, metastasis, and chemotherapy resistance [101]. In gastric cancer (GC), exosomal DACT3-AS1 derived from CAFs inhibited cell proliferation, migration, and invasion by targeting miR-181a-5p/sirtuin 1 (SIRT1) axis, and increased the sensitivity of GC cells to chemotherapy drugs through the SIRT1-mediated ferroptosis [102]. Research on head and neck cancer (HNC) has revealed that CAFs-derived exosomes contained lower levels of miR-3188 compared to normal fibroblasts (NFs)-derived exosomes, and exosomal miR-3188 from CAFs inhibited HNC tumour growth by targeting BCL2 [103]. Studies on oral squamous cell carcinoma (OSCC) have demonstrated that CAFs-derived exosomal miR-34a-5p was transferred to OSCC cells. By binding to its direct downstream target AXL, miR-34a-5p promoted EMT-mediated metastasis of cancer cells by inducing through AKT/GSK-3β/β-catenin signalling pathway [104] (Fig. 1).

## MSCs-derived exosomes

MSCs are pluripotent stromal cells that exist in various tissues. MSCs have low immunogenicity, immunomodulatory ability, easy cultivation and expansion in vitro, and the ability to migrate to tumours or inflammatory sites [105]. Therefore, they have been widely used in experimental and clinical studies of inflammation and tumour diseases. MSCs can produce a large number of exosomes under resting and stress conditions. MSCs-derived exosomes (MSCs-Exo) have similar biological functions as MSCs and are vital factors in mediating the exchange and transmission of intercellular information. MSC-Exo has complex and bidirectional effects on tumours, including tumour growth, metastasis, invasion, angiogenesis, and drug resistance. MSC-Exo can promote tumour growth and metastasis, as well as inhibit tumour occurrence and development [106, 107].

MSC-Exo can activate specific cellular signalling pathways and affect the occurrence and development of tumours by stimulating protein molecules. Research has shown that bone marrow mesenchymal stem cell-derived exosome (BMSC-Exo) reduced the expression of cyclin E1 (CCNE1) and CCNE2 through miR-144, thereby inhibiting the proliferation, colony formation, and S-phase arrest of NSCLC cells, preventing normal DNA synthesis and indirectly inhibiting tumour growth [108]. MSC-Exo mainly regulated the corresponding signalling pathways by promoting the delivery of certain specific miRNAs (such as miR-1587, miR-208a, etc.), thereby regulating the synthesis of certain proteins and promoting tumour metastasis and invasion [109, 110]. MSC-Exo can also promote tumour metastasis and invasion by delivering cytokines (such as IL-6) to target cells [111]. MSC-Exo was rich in angiogenic factors that can regulate tumour angiogenesis [107]. Studies have shown that MSC-Exo activated the extracellular signal-regulated protein kinase 1/2 (ERK1/2) signalling pathway or upregulated the expression of proliferating cell nuclear antigen (PCNA), accelerated tumour angiogenesis, and further promoted tumour development [112]. However, researchers have also demonstrated that MSC-Exo indirectly inhibited vascular endothelial growth factor through miRNA, thereby reducing tumour angiogenesis [113] (Fig. 1).

## Tumour-derived exosomes

Tumour cells produce exosomes in large quantities and use these exosomes to transfer oncogenes to other cells and change their original environment to establish a microenvironment suitable for their own metastasis and rooting in advance. In addition, TDEs also transmit signalling factors that inhibit the immune response to tumours, thereby reducing the immunity of tumour patients, especially those in advanced stages [114]. TAMs and exosomes are crucial in the formation of the premetastatic niche [115]. Exosomal miR-934 derived from colorectal cancer (CRC) regulates the formation of premetastatic niches and promotes liver metastasis by inducing macrophage M2 polarization [116] (Fig. 1g). TDEs secreted by primary tumour cells could induce tissue resident interstitial macrophages (IM) in the premetastatic niche to upregulate the immunosuppressive molecule programmed death ligand 1 (PD-L1) and secrete a large amount of lactic acid, thereby establishing an immunosuppressive microenvironment to promote solid tumour metastasis [117]. Studies have found that TDEs containing miR-424 secreted by CRC cells can be taken up by infiltrating immune cells in the TME, which can reduce the expression of CD28 and CD80 in T cells and DCs, destroy the costimulatory signal transduction of T cells, and lead to the failure of T cells to fully activate the antitumour response, causing resistance to immunotherapy in patients [118] (Fig. 1g).

Exosomes secreted by tumour cells have specific antigens on their surface or carry some of the genes and secretions of the source cells. These “special” exosomes communicate information and transfer substances with immune cells and stimulate immune cells to change their physiological state and function to activate downstream immune responses, promote the secretion of anti-inflammatory factors, and inhibit tumour cell proliferation [119]. Studies showed that murine bladder cancer (BC) cell-derived immunogenic exosomes inhibited murine bladder MB49 tumour growth by CD8^+^ T-cell cytotoxicity and immune cell infiltration into the tumours [120] (Fig. 1g).

## Different substances carried by exosomes

**Fig. 2:**
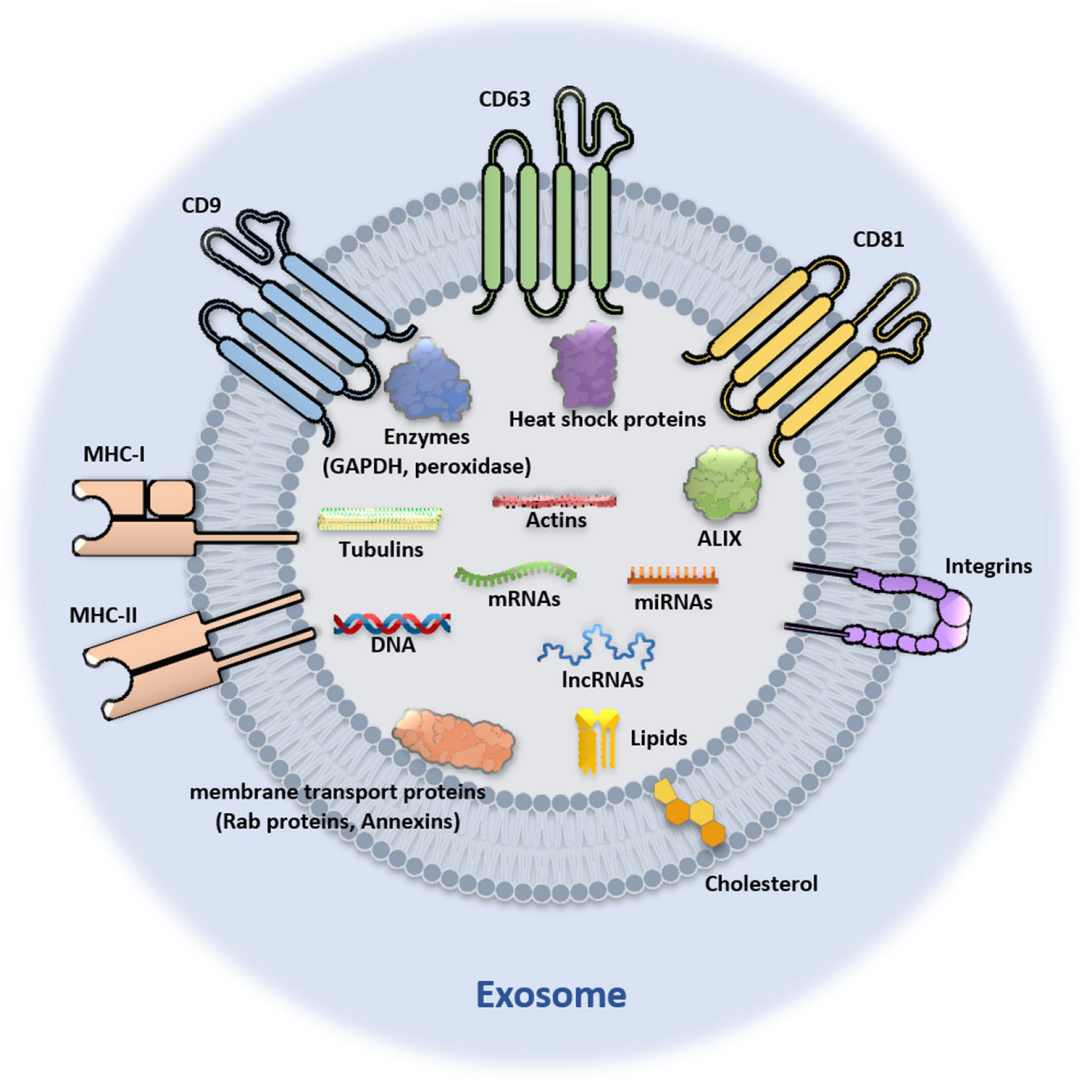
Different substances carried by the exosomes. Exosomes in various organisms and cells carry proteins, lipids, RNAs, and DNA.

Exosomes in various organisms and cells contain a total of 9769 kinds of proteins, 1116 kinds of lipids, 3408 kinds of mRNAs and 2838 kinds of miRNAs based on the exosome database [121]. Protein content is largely dependent on the cellular origin of exosomes and is dominated by certain molecular types, such as localized proteins and fusion proteins (e.g., tetratransmembrane proteins (e.g., CD9, CD63, CD81, CD82, etc.), lactadherin, integrin, etc.), cytoplasmic enzymes (e.g., GAPDH, peroxidase, pyruvate kinase, lactate dehydrogenase, etc.), molecular chaperones (such as heat shock proteins: HSP60, HSP70, HSP90, and other and small heat shock proteins), membrane transport proteins (such as Rab proteins, ARF GTPase and annexin), cytoskeletal proteins (such as actin and tubulin), proteins related to exosome formation, such as tumour susceptibility gene 101 protein (TSG101), ALG-2 interaction protein X (ALIX), endosomal sorting complex required for transport (ESCRT), and signal transduction proteins (such as protein kinases and heterotrimer G proteins) [30, 122]. In addition, exosomes also contain a variety of specific proteins related to cell origin and tissue type, such as major histocompatibility complex I (MHC-I), MHC-II, and cell adhesion molecules. Lipids are also the main components of exosomes, which are rich in cholesterol, glycerol, glycerophospholipids, phospholipids, sphingolipids and glycosylceramides (including sphingolipids and ceramides) [123]. In addition to proteins and lipids, exosomes contain numerous functional RNA molecules (mRNAs and noncoding RNAs, such as long noncoding RNAs (lncRNAs) and miRNAs) and DNA molecules [30] (Fig. 2). These components interact with each other in the TME and play important roles in the occurrence and development of tumours.

## The mechanism by which exosomes affect immunotherapy for solid cancers

MDSCs are groups of heterogeneous cells derived from bone marrow that have the ability to significantly inhibit immune cell responses [124, 125]. Studies have shown that tumour-derived exosomal HSP72 stimulates the expansion of MDSCs through Erk and activates STAT3 by inducing autocrine IL-6 in a TLR2/MyD88-dependent manner. Inhibiting the secretion of exosomes using the exosome inhibitor dimethylamine in three different mouse solid tumour models enhanced the antitumour effect of cyclophosphamide [126].

Innate immune cells, especially macrophages, play important roles in tumour metastasis. Macrophages not only promote the occurrence and development of tumours but also play vital roles in the establishment of an immunosuppressive microenvironment premetastatic niche [127]. Studies have shown that the M2 polarization of macrophages induced by CRC cell-derived exosomes containing miR-934 could be mediated by the CXCL13/CXCR5/NF-κB/p65 axis to promote colorectal cancer liver metastasis (CRLM), which led to crosstalk between tumour cells and TAMs, creating an inflammatory microenvironment for CRLM [116]. Another study revealed that TDEs increased PD-L1 expression in macrophages and polarized macrophages into an immunosuppressive phenotype through NF-κB-dependent glycolytic dominant metabolic reprogramming [117]. TDEs induced an increase in glucose uptake via TLR2 and NF-kB and inhibited mitochondrial oxidative phosphorylation by increasing NOS2 levels, thereby promoting the conversion of pyruvate to lactate, which fed back on NF-kB and further elevated PD-L1 levels [117].

T-cell exhaustion is a state of diminished function characterized by a gradual loss of T-cell effector function and self-renewal [128]. T-cell exhaustion is considered to be one of the pathways of drug resistance in cell therapy [129]. Sphingosine-1-phosphate (S1P), which is catalysed by sphingosine kinase-1 (SPHK1) through the phosphorylation of sphingosine, is highly expressed in some solid tumours and is related to proliferation, migration, invasion, adhesion, and angiogenesis [130, 131]. Studies have demonstrated that SPHK1-packaged exosomes can upregulate S1P in the ovarian cancer microenvironment, which induces immune suppression through T-cell exhaustion and increases PD-L1 levels via E2F1-mediated transcription [132].

T lymphocytes are important parts of the tumour immune microenvironment, including cytotoxic T cells, Tregs and Th17 cells. Treg/Th17 cell imbalance is associated with multiple diseases, such as systemic lupus erythematosus, diabetes, and tumours [133]. Studies have shown that exosomes derived from TAMs transport miR-29a-3p and miR-21-5p to CD4^+^ T cells, leading to Treg/Th17 imbalance and inducing EOC tumour growth and metastasis by directly targeting STAT3 [72]. Tregs inhibited the activity of CD8^+^ T cells. An increase in the proportion of Treg/CD8^+^ T cells in tissues is significantly associated with poor prognosis of patients [134]. Studies have shown that DCs with high expression of the immunosuppressive receptor CD300A can inhibit the tissue invasion of Tregs. The transplanted melanomas in the CD300A-deficient mice were larger and grew faster. IFN-β secreted by DCs was the main driving factor regulating the tumour invasion of Tregs. They demonstrated that tumour-derived exosomes promoted the secretion of more IFN-β by DCs. In addition, compared with normal DCs, CD300A-deficient DCs produced more IFN-β after being treated with tumour-derived exosomes, thus increasing the tumour invasion of Tregs [135].

Cancer-associated lymphatic endothelial cells (LECs) are one of the components of the TME that can help tumour metastasis and immune escape. In addition to the high expression of PD-L1 in various cancers and host hematopoietic cells, PD-L1 expressed by some nonhematopoietic cells is also involved in the immune response. For example, PD-L1 expressed by LECs can inhibit T-cell activity in vitro [136]. MiR-1468 was highly expressed in exosomes secreted by cervical cancer cells. When human dermal lymphatic endothelial cells (HDLECs) are treated with exosomes, the expression of PD-L1 in HDLECs is increased, exhibiting a tumour-associated phenotype of lymphatic endothelial cells, thereby suppressing tumour immunity. These tumour-associated lymphatic endothelial cells could inhibit the secretion of various antitumour factors by CD8^+^ T cells and promote apoptosis of CD8^+^ T cells, thereby reducing the antitumour effects and promoting the growth of cervical cancer cells. They also found that after exosomal miR-1468 was taken up by lymphatic endothelial cells, it directly targeted the inhibition of the HMBOX1 transcription factor, causing a decrease in the transcription level of its downstream gene SOCS1, thereby attenuating the inhibitory effects of SOCS1 on the JAK/STAT3 signalling pathway. Ultimately, by activating this pathway, the expression of PD-L1 is increased, inducing the formation of tumour-associated lymphatic endothelial cells [137].

CD28-CD80/86 costimulatory signals are critical for the response to immune checkpoint inhibitor therapy. Some studies have shown that the effectiveness of anti-PD-1 is partly due to the recovery of CD28-CD80/86 signals [138]. High levels of miRNA-424 in exosomes secreted by colon cancer cells. In vivo experiments demonstrated that injection of colon cancer-derived exosomes could inhibit the activity and expansion of tumour-specific T cells and DCs, reduce the expression of CD28 and CD80/86, and promote tumour growth. However, exosomes derived from tumour cells transfected with miRNA-424 inhibitor did not inhibit the activities of T cells and DCs and lost the role of promoting tumour growth. The modified exosomes combined with immune checkpoint inhibitor therapy (ICBT) could significantly increase sensitivity to ICBT [118].

## Effects of exosomes on different treatment methods of immunotherapy for solid cancers

### Effects of exosomes on adoptive cell therapy

CAR-T cells have achieved significant performance in the treatment of some malignant tumours of the hematopoietic system, but they are still ineffective in most solid tumours [139]. Factors leading to poor efficacy in solid tumours include limitations of expansion and persistence, such as poor infiltration of CAR-T cells in the TME, exhaustion, and insufficient antigen [140]. Recently, researchers reported that CAR-T cells expressing the immunostimulatory RNA RN7SL1 could enhance immune efficacy in a melanoma mouse model losing CAR antigen by improving autonomous CAR-T-cell function and promoting endogenous immunity by preferential uptake of RN7SL1-containing exosomes by innate immune cells rather than tumour cells [140]. Studies have shown that exosomes containing CAR expressed high levels of cytotoxic molecules, which could significantly inhibit tumour growth [82]. Further experiments revealed that CAR exosomes did not express PD-1 compared to CAR-T-cell therapy, and treatment with recombinant PD-L1 did not impair the antitumour effect of CAR exosomes. Experiments conducted by researchers in an in vivo preclinical model of cytokine release syndrome have shown that the use of CAR exosomes was safer than CAR-T therapy [82]. Likewise, another study on CAR-T-cell-derived exosomes revealed a stronger penetration capacity and milder CRS for HER-2-expressing target solid tumour cells compared with their parental CAR-T cells [141]. However, exosomes derived from SH-SY5Y neuroblastoma cells significantly impacted CD4^+^ CAR T-cell efficacy in a preclinical setting by weakening the tumour cytotoxicity of CD4^+^ CD171-specific CAR T cells [142]. In summary, exosomes derived from CAR-T cells enhanced the immune efficacy of CAR-T-cell therapy, whereas exosomes derived from tumour cells weakened the immune efficacy of CAR-T-cell therapy.

### Effects of exosomes on ICIs therapy

The discovery of ICIs anti-PD-1/anti-PD-L1 is a milestone in the course of human fighting against cancers, which effectively improves the survival of patients with various advanced cancers [143]. However, due to the occurrence of primary and secondary resistance, the clinical response rate of anti-PD-1/PD-L1 treatment is not high, only 15-30% [144]. Currently, little is known about the mechanism of resistance to PD-1/PD-L1 therapy. TDEs contain high levels of PD-L1, which can inhibit the systemic immune response [145]. Studies have reported that the PD-L1 level of TDEs is negatively correlated with the outcome of anti-PD-1/PD-L1 treatment, indicating that TDEs are involved in the development of resistance to anti-PD-1/PD-L1 treatment [146–148]. Studies have shown that PD-L1 also exists on the surface of exosome membranes released by melanoma cells, and its content is much higher than that on the surface of melanoma cell membranes. In addition, PD-L1-rich exosomes secreted by melanoma cells could also enter the peripheral vascular system of the body and contact T cells, inhibiting their functions at the same time to achieve the purpose of comprehensively inhibiting T-cell function, thus extending PD-L1-mediated tumour immunosuppression to the whole body [146]. Likewise, studies on other kinds of solid tumours revealed that exosomal PD-L1 inhibited antitumour effects, and the mechanism of distal inactivation by gene blocking enhanced the activity of T cells in draining lymph nodes and induced systemic antitumour immunity and memory. The results from prostate cancer models and colon cancer models suggested that inhibiting the release of PD-L1 exosomes overcame the insensitivity to ICIs in a large proportion of patients [147]. Studies have shown that PD-L1-positive exosomes secreted by tumours in the TME inhibit the killing effect of T cells on breast cancer cells and promote the growth of tumours, suggesting that the combination of inhibitor exosome secretion and anti-PD-L1 antibodies in the clinical treatment of tumours is helpful to improve the antitumour immune response and eliminate tumours [149]. TDEs could compete with tumour cells to bind to anti-PD-L1 through PD-L1 in vitro [150]. With the increase or decrease in TDEs in solid tumour mouse models, the therapeutic effects of anti-PD-L1 were inhibited or enhanced, respectively. They also found that the distribution of anti-PD-L1 binding to TDEs changed significantly in vivo, making it easier to be phagocytized by tissue macrophages and peripheral blood monocytes, thereby entering the lysosomal degradation pathway faster [150].

CD47 is an immune checkpoint that is typically highly expressed on tumour cells, and inhibits macrophage phagocytosis by binding to signal regulatory protein alpha (SIRPα) on phagocytes [50]. Block CD47/SIRPα interaction has been used as a therapeutic strategy, including monoclonal antibodies targeting CD47 or SIRPα and recombinant SIRPα protein for antagonizing interaction [151]. Nevertheless, these therapeutic strategies have some drawbacks. For instance, the limited specificity caused the binding of the anti-CD47 antibody to non-tumour cells, resulting in anaemia and leukopenia. Furthermore, although the use of recombinant SIRPα protein enhanced phagocytosis in vitro by blocking inhibitory signals, the therapeutic effect in vivo can only be achieved by combining with other anticancer drugs [152]. In order to overcome these limitations, researchers developed an exosome-based immune checkpoint blocker, i.e., exosomes containing SIRPα variants (SIRPα-exosomes) on their surface, which significantly improved their affinity for CD47 [153]. The macrophage phagocytosis for tumours was enhanced after intravenous administration of SIRPα-exosomes, and an effective antitumour T-cell response was induced, suggesting that SIRPα-exosomes had great potential to block immune checkpoints.

### Effects of exosomes on cytokine therapy

Cytokines are mainly produced by tumour cells and immune cells, and are types of protein or micro-molecule polypeptides with immune regulation and effector functions, playing vital roles in the human immune response. Interleukins and related cytokines are the means of communication between innate and adaptive immune cells, as well as non-immune cells and tissues. Therefore, cytokines play crucial roles in the occurrence and development of cancers. In addition, cytokines are effective immune regulatory protein molecules that stimulate the function, survival, and proliferation of natural NKs and T cells, thereby mediating immune responses against tumours [154, 155]. Cytokines are crucial mediators of signal transduction in the TME, with multiple effects in promoting and inhibiting tumours. Cytokines, as key elements regulating TME-tumour immune cell interactions, play pivotal roles in antitumour immunotherapy [156]. Primary tumours can release cytokines, which activate distant tissues and create a pre-metastatic niche for circulating tumour cells to support metastatic growth [157]. Exosomes secreted by cancer cells are the basic mediators for the formation of the pre-metastatic niche, which fosters intercellular communication and reprogramming of TME [158]. Researchers found that exosomes from breast cancer patients contained higher levels of CCL2 and IL-6 [159]. Moreover, the pure exosomes isolated from EO771 BC cells cultured in vitro largely lack cytokines and growth factors. Repeated injection of tumour interstitial fluid (TIF) exosomes altered the immune cell composition of the lungs, spleen, and liver: decreases of NK cells in both lungs and spleen; an increase of G-MDSCs in lungs; s decrease of DCs in the liver; an increase of Ly6C^lo^ macrophages. The above results indicated a correlation between cytokines in TME and TDEs, which altered the immune landscape of distant organs and fostered the metastasis burden of BC.

### Effects of exosomes on oncolytic virus therapy

Oncolytic viruses (OVs) are types of viruses that selectively replicate in tumour cells and cause tumour cell apoptosis while preserving normal tissue from destruction. OVs exert antitumour effects through various pathways, including direct oncolysis, induction of innate immune response to kill tumours, induction of adaptive immune response to kill tumours, alteration of TME, disruption of blood supply to tumours, etc. [160, 161]. ICIs and CAR-T therapy are currently mainly limited to hematomas due to poor permeability to solid tumours, while OVs can complement ICIs and cell therapy drugs. OVs increase T cell migration to tumours, enhance T cell survival and expansion, enhance antigen-presenting cells (APCs) function, reverse T cell exhaustion, and turn cold tumours into hot tumours, thereby improving the efficacy of ICIs and cell therapy drugs [162]. Researchers have discovered new artificial microRNAs (amiRNAs) that significantly promoted the tumour-killing effects of oncolytic viruses and small molecule therapies [163]. They found that tumour cells infected with oncolytic viruses secreted increased exosomes. Through a series of experiments such as exosome isolation, identification, and tracing, they found that when tumour cells were infected with oncolytic viruses carrying amiR-4, the exosomes secreted by the cells contained amiR-4 and could be transmitted to other cells that were not infected with oncolytic viruses [163]. Furthermore, studies have shown that exosomes selectively deliver antitumour drugs to tumour tissue, thereby reducing the side effects of chemotherapy. In addition, the use of exosomes to load drugs did not alter the inflammatory response associated with NF-κB induced by oncolytic viruses, as well as the numbers of CD3^+^, CD4^+^, and CD8^+^ T cells infiltrating the tumour, providing strong supporting evidence for exosomes as drug carriers [164].

### Effects of Exosomes on cancer vaccines therapy

Due to their good biocompatibility, convenient storage and transportation, ability to reduce drug dosage by increasing the stability of encapsulated drugs, and ability to generate active targeting through surface modification, exosomes have broad prospects for tumour treatment. The use of exosomes for vaccine preparation is a new direction in tumour immunotherapy. The surface of TDEs can load MHC-I and tumour antigens, effectively inducing the reaction of T lymphocytes, and can be developed into tumour vaccines [165]. It was confirmed that the antitumour effects of TDEs in vivo largely depended on DCs. The direct interaction between exosomes and immune cells usually manifests as immunosuppression, while the direct interaction between Dex and immune cells is mostly immunoactivation. Moreover, due to the noncellular structures, Dex has advantages over cellular dendritic cell vaccines in terms of easy preparation, quality control, and mass production. Therefore, Dex is more ideal carrier of tumour vaccines [166]. Researchers have designed a novel nanovaccine platform that used Dex as a carrier to load patient-specific neoantigens for personalized immunotherapy [63]. The Dex delivery system has a good storage effect and targeting ability toward lymph nodes at the injection site, successfully inducing antigen-specific T-cell and B-cell-mediated immune responses in mice. The neoantigen-exosome nanovaccine system has been applied in B16F10 melanoma and MC-38 colon cancer mouse models. By stimulating multiple immune responses, the system significantly inhibited the growth of various tumours, prolonged mouse survival, delayed tumour occurrence, established long-term immune memory protection, and effectively eliminated lung metastasis. Mechanism studies have shown that the neoantigen-exosome nanovaccine fostered the release of various antitumour immune factors, stimulated more antitumour T cells and immune infiltration to the tumour site, and exerted an effect on clearing the tumour. At the same time, it activated B cells to secrete a large number of antigen-specific antibodies, achieving a synergistic effect of humoral and cellular immunity [63]. Apart from Dex-based vaccines, exosomal vaccines from antigen-stimulated M1-type macrophages and outer membrane vesicles (OMVs)-based vaccines also displayed antitumour effects in a wide variety of solid tumours [167, 168].

## Exosomes influence immunotherapy by remodelling the TME

The dynamic network of nonmalignant cells, noncellular components, signalling molecules, and ECM around a tumour is called the TME. There is a two-way interaction between the TME and tumours, which maintains and promotes tumour growth and proliferation. Studies have shown that the TME plays a key role in tumour progression, including its positive role in inhibiting tumour occurrence and progression at the early stage of carcinogenesis and the great potential of “reprogramming” the TME at the late stage of cancer treatment. Studies have proven that exosomes can influence immunotherapy by remodelling the TME. Studies have shown that TAM-derived exosomes remodel the TME through the transfer of microRNAs, resulting in an imbalanced Treg/Th17 ratio of T-cell subsets in the microenvironment of EOC, which generates an immunosuppressor microenvironment and ultimately promotes the growth and metastasis of EOC tumour cells [133].

γδ T cells are a special group of lymphocytes with antitumour or tumour-promoting functions and are activated in an MHC-dependent manner [169]. These MHC-limiting and costimulation-dependent characteristics make γδ T cells good candidates for effective tumour immunotherapy. Studies have found that γδ T cells isolated from patients with different solid tumours can effectively kill tumour cell lines or primary tumour tissue [170–173]. Normoxic exosomes in oral squamous cell carcinoma (OSCC) stimulate γδ T-cell expansion in a dendritic cell-dependent manner. Hypoxic exosomes did not have the ability to stimulate γδ T-cell activity but enhanced the inhibitory effects of MDSCs on γδ T cells through the miR-21/PTEN/PD-L1 pathway [174].

Studies have revealed that higher infiltration levels of CD73^high^ TAMs in the head and neck squamous cell carcinoma (HNSCC) microenvironment are associated with poor prognosis. Exosomes derived from HNSCC cancer cells carried CD73 (sEVs^CD73^). sEVs^CD73^ could recruit macrophages and Tregs but led to a decreased number of infiltrated CD8^+^ T cells. sEVs^CD73^ activated NF-κB in the TAM pathway, thereby suppressing immune function by increasing the secretion of cytokines such as TGF-β1, IL-6, IL-10, and TNF-α. The absence of sEVs^CD73^ enhanced the sensitivity of anti-PD-1 therapy by reversing immune suppression [175].

## Advantages of exosomes penetrating the blood-brain barrier in the treatment of brain tumours

Glioma is the most common primary tumour in the human central nervous system. Among them, glioblastoma (GBM), with the highest degree of malignancy, is the most invasive and fatal. Although maximum surgical excision is the primary treatment measure, it cannot avoid postoperative recurrence [176]. Due to the existence of the blood-brain barrier (BBB) and blood-brain tumour barrier (BBTB), most drug candidates cannot effectively accumulate in glioma lesions [177]. In addition, the immunosuppressive microenvironment is also a major obstacle to the treatment of glioma [178]. Therefore, it is crucial to combine multiple strategies to overcome the therapeutic barrier of glioma. Immunotherapy works by mobilizing the body’s immune system to suppress and kill tumour cells. Oligodeoxynucleotides containing unmethylated cytosine-guanine motifs (CpG ODN) can stimulate DCs and TAMs in the GBM microenvironment to generate a series of antitumour immune responses [179, 180]. Researchers designed nanomicelles formed by the self-assembly of tanshinone IIA (TanIIA) and glycyrrhizic acid (GL) (TanIIA-GL nanomicelles, TGM) and loaded them onto exosomes derived from serum. Serum-derived exosomes had abundant transferrin receptor expression, which could target the brain by binding to free transferrin in the blood. The immunoadjuvant CpG ODN 1826 was modified on the surface of exosomes to construct a biomimetic nanodelivery system integrating glioma targeting, chemotherapy and immunotherapy, named CpG-EXO/TGM. CpG-EXO/TGM could significantly stimulate the maturation of DCs, promote immature T cells to differentiate into CTLs, and promote M2-type TAMs to reverse polarization into M1-type TAMs. At the same time, they secrete a large amount of antitumour-related factors, inducing antitumour immune effects. After the recurrence model of GBM was established, CpG-EXO/TGM activated immune memory cells in mice, reactivated the immune response, and prevented GBM recurrence in vivo. Meanwhile, compared with the EXO/TGM group and normal saline group, the median survival time of mice in the CpG-EXO/TGM group was significantly longer, the body weight had no effect, and no obvious toxic side effects were observed [181].

## Exosomes influence immunotherapy by remodelling the lymph node microenvironment

**Fig. 3:**
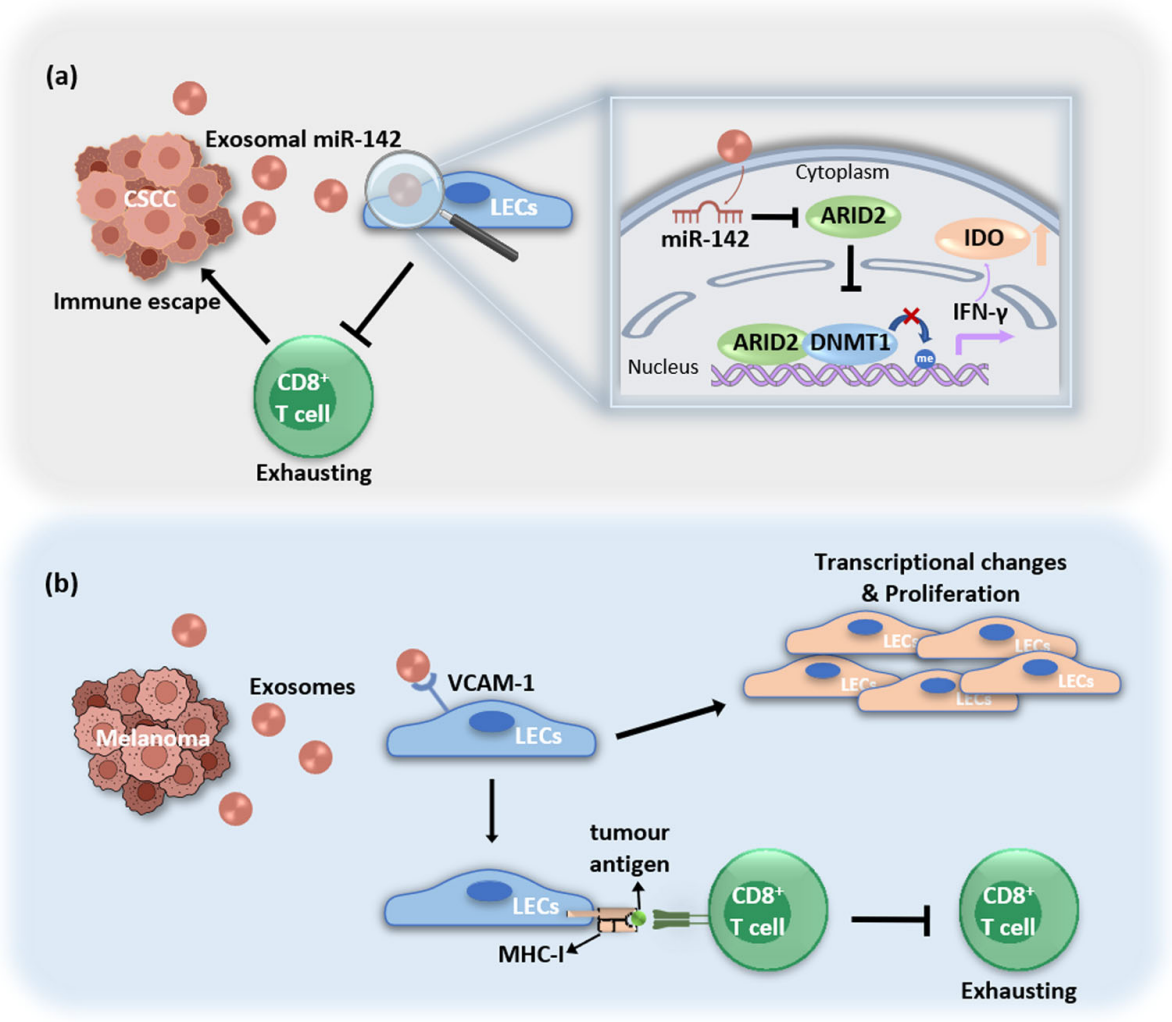
Exosomes influence immunotherapy by remodelling the lymph node microenvironment. **a** The transfer of miR-142-5p secreted by CSCC to LECs led to downregulation of ARID2 expression and inhibition of DNA methyltransferase 1 (DNMT1) recruitment to the IFN-γ promoter, enhancing IFN-γ transcription by inhibiting promoter methylation, which led to an increase in IDO activity, thereby exhausting CD8^+^ T cells and improving the immune escape of cancer cells. **b** Melanoma-derived exosomes induced transcriptional changes and proliferation of LECs, as well as apoptosis of antigen-specific CD8^+^ T cells, by promoting the transfer of tumour antigens to LECs and cross-presenting tumour antigens on MHC-I.

The clinical efficacy of immunotherapy is closely related to the immunosuppressive state of the TME and is affected by the dynamic interaction between tumour cells and LECs. Researchers have revealed that LECs, as an indispensable member of the TME immune checkpoint, may become a potential new target for the early diagnosis and treatment of cervical squamous cell carcinoma (CSCC) metastasis [182]. Researchers analysed the CSCC tissue microarrays of 116 patients using ISH and found that the expression of miR-142-5p was positively correlated with the number of indoleamine 2,3-dioxygenase (IDO)-positive lymphatic vessels (LVs) and total LVs around cancer and negatively correlated with the number of infiltrating CD8^+^ T cells. They found that exosomes secreted by CSCC have typical exosomal characteristics, and miR-142-5p participates in crosstalk between CSCC cells and HDLECs through the exosomal pathway. The transfer of miR-142-5p secreted by CSCC to LECs led to downregulation of ARID2 expression and inhibition of DNA methyltransferase 1 (DNMT1) recruitment to the IFN-γ promoter, enhancing IFN-γ transcription by inhibiting promoter methylation, which led to an increase in IDO activity, thereby exhausting CD8^+^ T cells and improving the immune escape of cancer cells [182] (Fig. 3a).

The tumour-draining lymph node (tdLN) is the portal for tumour cell metastasis and the initial site for the adaptive immune response. Although a large number of DCs and lymphocytes were enriched in the draining lymph nodes, continuous excessive tumour secretion stimulation and Treg accumulation, which led to immune tolerance, inhibited DC maturation and activation and amplification of effector T cells and failed to produce effective antitumour effects, leading to tumour cell escape and metastasis [183, 184]. Researchers found that the highly selective interaction between melanoma-derived exosomes and some macrophages and LECs in the tdLNs contributed to the formation of a premetastatic niche and suppression of tumour immunity. Melanoma-derived exosomes could induce apoptosis of antigen-specific CD8^+^ T cells by promoting the transfer of tumour antigens to LECs in tdLNs and cross-presenting tumour antigens on MHC-I [185] (Fig. 3b).

## The mechanisms of exosomes affecting immunotherapy in different solid cancers

**Fig. 4:**
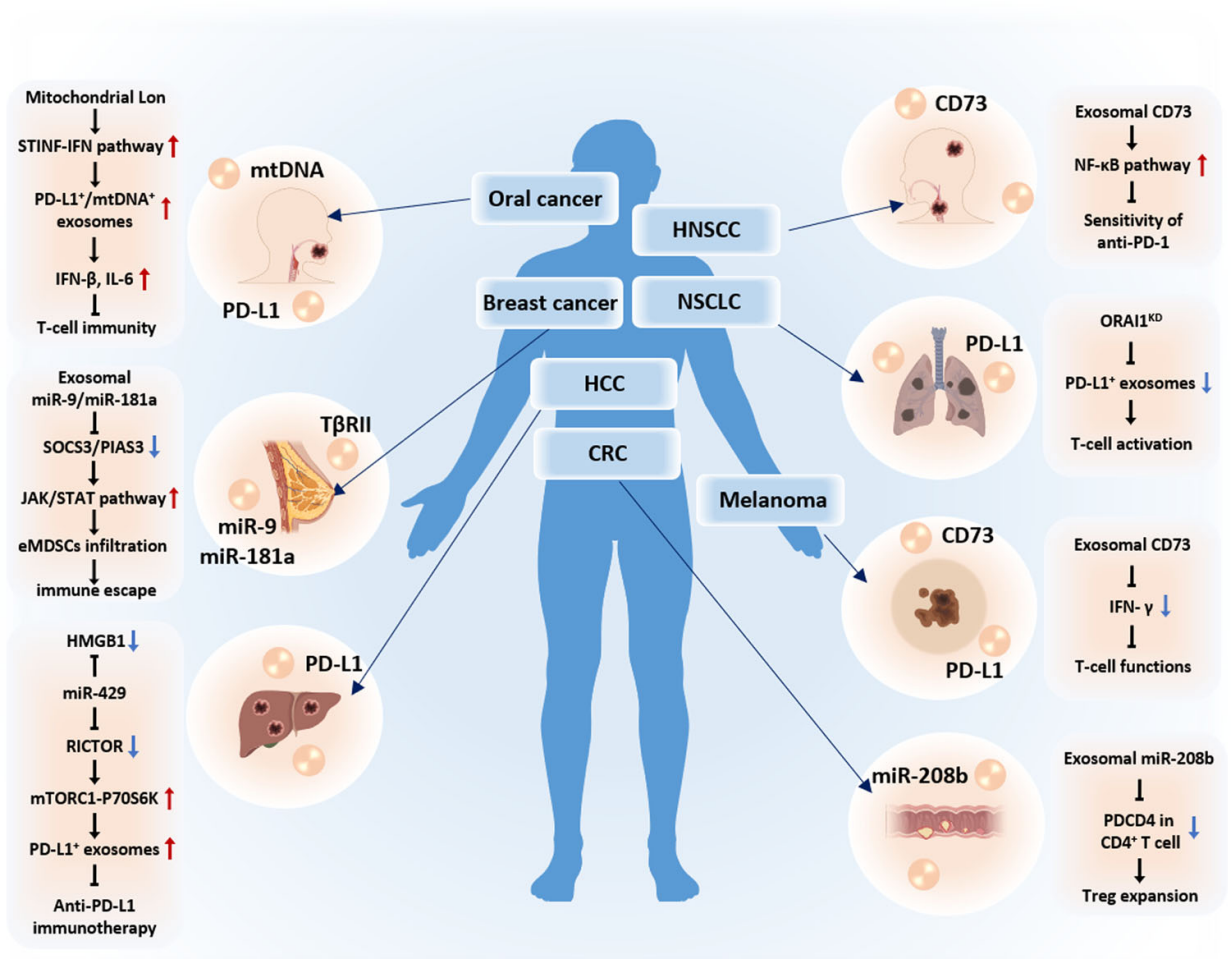
The mechanisms by which exosomes affect immunotherapy for different solid cancers.

Cancer cells release more exosomes than healthy cells, mainly due to the activation of oncogenes, indicating a potential relationship between tumours and exosomes. Exosomes are closely related to tumours and can affect tumour cells through various mechanisms in their occurrence, development, and treatment. Due to the diversity of exosomes released by tumour cells, exosomes have been widely studied in various types of solid cancer (Fig. 4).

Studies have shown that CD73 is expressed on isolated exosomes in the serum of patients with melanoma. The exosomes reduced IFN-γ release and inhibited the function of T cells. In the early stages of treatment, patients with strong drug responsiveness had an increase in serum exosomal PD-L1, while nonresponders had an increase in exosomal CD73. Therefore, they concluded that in the early stage of treatment, increased expression of CD73 in exosomes may lead to the tolerance of melanoma patients to anti-PD-1 drugs [186]. In a study of HNSCC, exosomal CD73 derived from HNSCC cancer cells weakened the sensitivity of anti-PD-1 therapy by contributing to immunosuppression through activating the NF-κB pathway in TAMs [175]. In studies on NSCLC, researchers found that the ORAI1 calcium channel could regulate intracellular calcium concentration and affect the secretion of exosomes carrying PD-L1 immune molecules produced by tumour cells. In tumour animal models, knocking down the ORAI1 channel in tumour cells inhibited the secretion of PD-L1 exosomes by tumour cells, increased the number of CD8^+^ cells in the spleen and tumour-infiltrating CD8^+^ cells, and impeded tumour progression [187]. In a study on hepatocellular carcinoma (HCC), the high-mobility group box 1 gene (HMGB1) and the important molecule RICTOR in the mTOR pathway competitively bound to members of the miR-200 family, especially miR-429. The interaction between the two at the RNA level promoted glutamine metabolism and malignant proliferation, self-renewal, and tumorigenesis of HCC cells. Moreover, this regulatory form could also inhibit the efficacy of anti-PD-L1 immunotherapy for HCC by promoting the production of mTORC1-P70S6K-dependent PD-L1^+^ exosomes [188]. A study on oral cancer reported that Lon regulated the metabolism of mitochondrial DNA (mtDNA) and the production of mitochondrial reactive oxygen species (ROS), whose upregulation induced cell secretion of extracellular vesicles carrying mtDNA and PD-L1 by the STING-IFN signalling pathway. Lon-induced exosomes further promoted immunosuppressive cytokine secretion from M2 macrophages, thereby weakening innate immunity and CD8^+^ T-cell immunity in the TME, ultimately promoting tumour development [189]. In a study on melanoma, it was demonstrated that HRS related to exosome biogenesis could be phosphorylated at the S345 site by ERK. P-HRS mediated the enrichment of PD-L1 in exosomes and secreted it to bind to the extracellular matrix around tumour cells, thereby inhibiting the infiltration of CD8^+^ T cells and achieving immunosuppressive effects [190] (Fig. 4).

Studies have reported that exosomes derived from highly metastatic triple-negative breast cancer (TNBC) cells carry active TGF-β type II receptor (TβRII) to stimulate TGF-β signalling in receptor cells. Exosomal TβRII could activate SMAD3 in CD8^+^ T cells and exert a synergistic effect with the transcription factor TCF1 to exhaust CD8^+^ T cells, ultimately hindering immunotherapy [191]. Studies on colon cancer reported that low levels of miR-208b in serum were associated with longer progression-free survival (PFS) in CRC patients. CRC cell lines could secrete miR-208b through exosomes to promote Treg expansion. MiR-208b could directly target and inhibit PDCD4 expression in CD4^+^ T cells to promote Treg differentiation, leading to immunosuppression in CRC mouse models [192]. A study on breast cancer revealed that miR-9 and miR-181a from tumour exosomes activated the JAK/STAT signal transduction pathway by targeting SOCS3 and PIAS3, respectively, thus promoting the expansion and infiltration of early-stage myeloid-derived suppressor cells in situ, inhibiting T-cell immunity, and promoting tumour growth and immune escape [193] (Fig. 4).

## Engineering exosomes for immunotherapy of solid cancers

Exosomes can mediate intercellular communication, possessing high physical and chemical stabilities and preferable biocompatibility. Therefore, they have been used as excellent carriers for a variety of biologically active compounds, such as nucleic acids, proteins, and nanomaterials [12]. Previous studies have shown that natural exosomes derived from tumour cells or immune cells could trigger antitumour activity and have the potential to become cancer vaccines [194]. However, for some reason, there are still hidden dangers in the clinical use of natural exosomes. For example, natural exosomes are easily trapped in nonspecific tissues (especially the liver and lung), resulting in insufficient targeting in vivo. The heterogeneity and complexity of natural exosomes affect the therapeutic effect, and efficient production and standardized monitoring are lacking [195]. Therefore, artificially modified engineered exosomes have become a new treatment strategy and are expected to become another option for exosome-based treatment [196]. Engineered exosomes have superior characteristics compared to natural exosomes, including tumour targeting, efficient intracellular delivery, high stability, long-term circulation, controllable drug release, and enhanced accumulation at lesion sites, significantly improving the safety, specificity, and effectiveness of exosome-based drugs for cancer therapy [197].

**Fig. 5:**
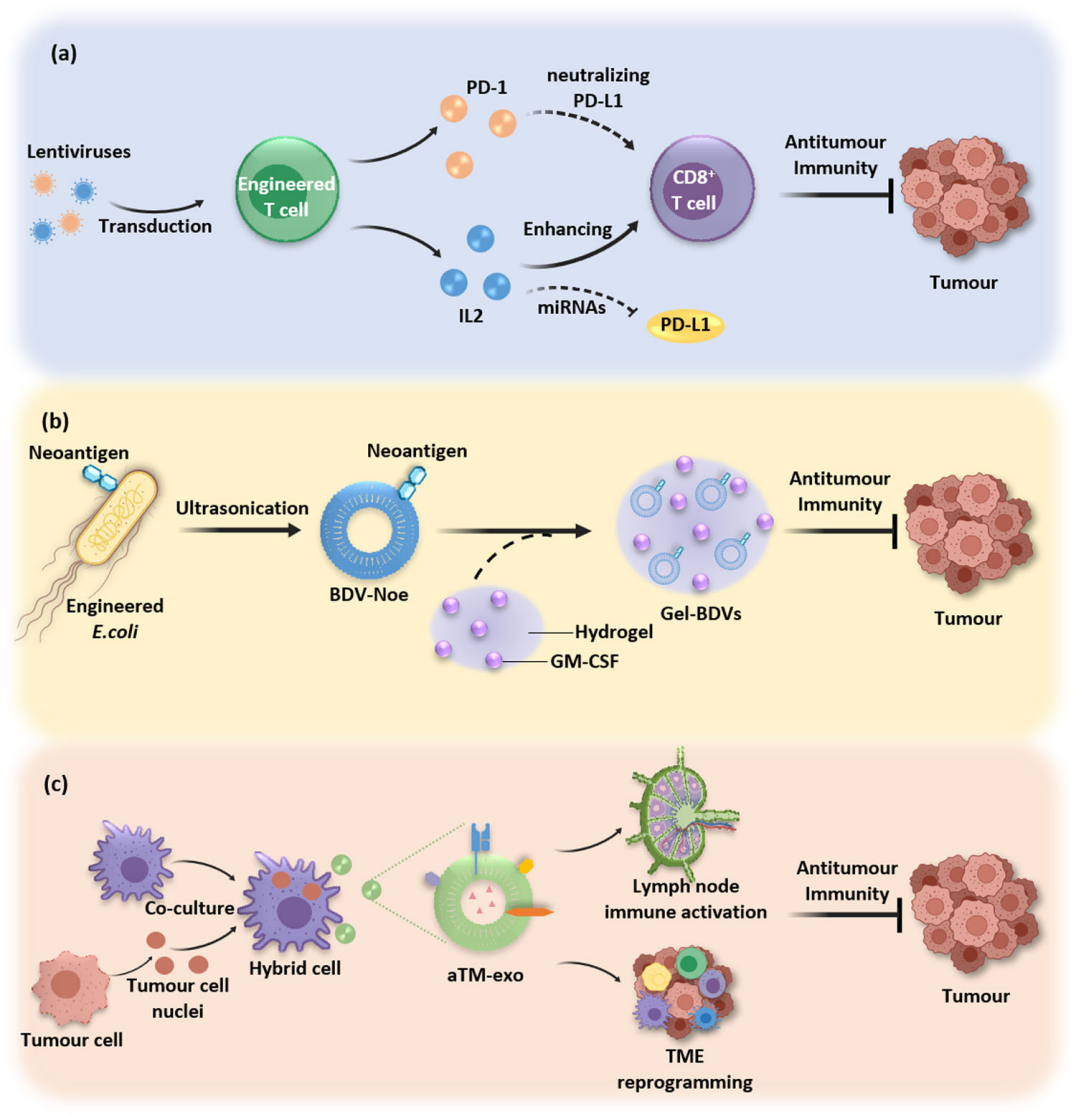
Engineering exosomes for immunotherapy of solid tumours. **a** Bioengineered T-cell-derived exosomes express PD-1 and IL2, which promote antitumour immunity. **b** OMVs are used in tumour drug delivery systems for antitumour immunity. **c** Macrophage-tumour chimeric exosomes activate the immune response and the tumour microenvironment for synergistic tumour immunotherapy.

Currently, the targeted function of exosomes is mainly achieved through biological fusion expression and chemical modification [198]. Researchers constructed T cells by transfection with GFP-PD-1 lentiviruses and demonstrated that exosomes derived from the constructed T cells expressed PD-1, which neutralized PD-L1 and reinvigorated tumour-infiltrating CD8^+^ lymphocytes in melanoma [199]. Studies demonstrated that exosomes derived from engineered Jurkat T cells that expressed IL-2 on the surface downregulated PD-L1 levels in melanoma cells by reprogramming miRNA levels and suppressed tumour development in melanoma-bearing immunocompetent mice [200] (Fig. 5a).

Currently, a class of lipid nanoparticles, such as liposomes, tumour cell membrane vesicles, and OMVs, have been widely used in tumour drug delivery systems [201]. Among them, OMVs, as exosomes naturally secreted by gram-negative bacteria due to their unique outer membrane budding form, have components similar to those of their parent bacteria, including lipopolysaccharide, outer membrane proteins, lipids, and nucleotides [202]. At the same time, OMVs also have the same size as pathogens and contain a variety of pathogen-associated molecular patterns and pathogen-associated antigens, which can effectively activate the innate immunity of the body [203]. A recent study reported engineering bacteria-derived exosomes called Synthetic Bacterial Vesicles (SyBV) with lysozyme and high pH treatment, possessing the advantages of avoiding systemic proinflammatory cytokine responses in mice compared to natural bacteria-derived exosomes [204]. Moreover, combined immunization with melanoma-derived exosomes and SyBV promoted tumour regression in mouse models of melanoma by balancing the production of antibodies and Th-1-type T-cell immunization [204]. Studies have reported that based on synthetic biology, specific neoantigens are synthesized and located on the bacterial outer membrane through genetic engineering methods, thereby achieving the presentation of neoantigens on the bacterial outer membrane [205]. By combining biomimetic bacterial-derived vesicles expressing neoantigens (BDV-Neo) with granulocyte-macrophage colony-stimulating factor (GM-CSF) using hydrogel as the packing materials, it was possible to continuously release GM-CSF, recruit DCs to obtain neoantigens, and activate T-cell immunity [205] (Fig. 5b). Researchers fused tumour antigens onto the surface of OMVs through genetic engineering [206]. As a natural medium for the interaction between intestinal flora and the immune system, OMVs can effectively penetrate the intestinal mucous layer and intestinal epithelial barrier and be ingested by antigen-presenting cells of the lamina propria, ultimately activating a strong antitumour immune response and immune memory effect in a mouse model of pulmonary metastatic melanoma and in mice bearing subcutaneous colon tumours [206].

Tumour immunotherapy can mobilize the body’s own immune cells to recognize and kill tumours, which is a promising new strategy for tumour treatment. Most of the existing tumour immunotherapy focuses on improving the function and number of immune cells in the body, but it is usually difficult to overcome the immunosuppressive microenvironment at the site of solid tumours, which makes it difficult for the infiltrating immune cells in the tumour to effectively perform their antitumour function [207]. Inspired by the immune activation effects of immune cell exosomes and the homing phenomenon of tumour cell exosomes, researchers developed lymph node-tumour dual-targeting exosomes for synergistic tumour immunotherapy [208]. Researchers introduced the nuclei isolated from tumour cells into activated macrophages to form hybrid cells and then used the hybrid cells to prepare biologically reprogrammed activated macrophage-tumour chimeric exosomes. These chimeric exosomes were equipped with various immune components, such as MHC-I molecules, costimulatory molecules, and immune-activated cytokines. With their nanosize and tumour homing molecules, these chimeric exosomes could flow into lymph nodes and accumulate in various types of animal tumour models, such as breast cancer and melanoma, significantly inhibiting the progression of primary tumours, metastatic tumours, and postoperative recurrent tumours [208] (Fig. 5c).

## Exosome vaccines for immunotherapy of solid cancers

In addition to the most concerning immunosuppressive agents, tumour immunotherapy also includes tumour vaccines, CAR-T cells and other immunocellular therapies, oncolytic viruses and immune factors [7]. Among them, tumour vaccines have unique advantages, including the ability to generate active immunity, long-term immunological memory, and synergistic effects of multitarget attack. Among various tumour vaccines, DCs exhibit excellent antitumour effects due to their powerful antigen-presenting ability, enabling DC vaccines to effectively induce antigen-specific immune responses. However, cellular vaccines face many problems, including a limited number of proliferations, complex quality control, limited source, and difficulty in production and preservation technology [209, 210]. Dex carry many key DC immune regulatory molecules. As noncellular vaccines, exosomal vaccines have the advantages of stable structure, relatively clear composition, high safety, easy preservation, suitability for mass production, and enhanced specificity of the immune response [165]. The researchers constructed an engineered exosome, HELA Exos, which is composed of a TLR3 agonist and ICD inducer and can activate dendritic cells in situ, specifically inducing immunogenic cell death (ICD) in breast cancer cells. The vaccines had effective tumour inhibitory effects in a xenograft mouse model with poor immunogenicity of TNBC and tumour organoids derived from human patients [166]. Researchers utilized biomimetic synthesis technology to construct an engineered DCs-derived exosomal vaccine platform, Antigen Self Presentation and Immunosuppression Reverse (ASPIRE). The ASPIRE vaccine system used DC membranes as a natural immunoactivated signal transduction vector, mediating the delivery of multiple costimulatory signals by regulating the expression of surface costimulatory molecules and ultimately achieving multidimensional antitumour immune activation. The ASPIRE system codelivered CD80/86 with an anti-PD-1 antibody, achieving effective reversal of tumour immune suppression and promoting functional remodelling of exhausted T cells in Lewis lung carcinoma (LLC) models and MC-38 colon carcinoma models [211]. Researchers developed a chimeric RNA exosome vaccine using A-Pas as a new antigen and DC-derived exosomes. In vivo experiments on animal models showed that chimeric RNA exosome vaccines could effectively inhibit tumour growth and significantly prolong the survival time of esophageal tumour mouse models. Further research found that a chimeric RNA exosome vaccine remodelled the TME by stimulating innate and adaptive immunity and played an immunotherapeutic role through a CD8^+^ T-cell-dependent mechanism [64].

Researchers extracted primary macrophages from mice and polarized them into the M1 type using lipopolysaccharide. Then, an engineered exosomal vaccine carrying antigens was prepared through “antigen feeding”. Studies have shown that therapeutic exosomal vaccines can enter tumour tissue and polarize M2 macrophages into M1 macrophages by downregulating the Wnt signalling pathway. The exosomal vaccines produced proinflammatory factors to kill tumour cells, solving the problem of TME immunosuppression. On the other hand, due to carrying tumour antigens, exosomal vaccines utilize the release of immune tolerance to further activate the body’s immune response and produce specific therapeutic antibodies. Both in vivo and in vitro experiments have shown that this system could utilize the therapeutic effect of exosomal vaccines to achieve the containment of malignant tumours and enhance the immunotherapeutic effect on B16-OVA-bearing mice [167].

Researchers used genetic engineering techniques to fuse one end of the polypeptide molecular glue onto the bacterial OMVs surface and bind the other end to the tumour antigen to build an individualized tumour vaccine that displayed tumour antigens on bacterial OMVs [168]. This OMVs-based vaccine provided efficient lymphatic drainage based on its size advantage and activated a variety of innate immune pathways, ultimately presenting a strong antitumour immune response in multiple preclinical solid tumour models [168]. In another study on melanoma, researchers utilized genetically engineered bacteria to synthesize tumour neoantigens and displayed them on the bacterial outer membrane. Then, BDV-Neo and GM-CSF were wrapped in the thermosensitive HP hydrogel to prepare a tumour vaccine. The vaccines sustainably released GM-CSF through subcutaneous injection, which recruited DCs to absorb BDVs and obtain new antigens, thereby promoting T-cell activation and differentiation into effector T cells. When combined with checkpoint blocking drug PD-1 antibody, the vaccines could effectively enhance the activity and proliferation of CD8^+^ cytotoxic T cells (CTLs), effectively recognizing and eliminating melanoma cells. In addition, tumour-specific T cells could be transformed into memory T cells to enhance the antitumour effect, effectively inhibit the relapse and lung metastasis of residual melanomas after surgery, and prolong survival [205]. Researchers have also designed an oral vaccine system based on bacteria robots working in vivo and loaded with tumour-specific antigens for tumour prevention and treatment. The oral vaccine system achieved immune stimulation by controlling genetically engineered bacteria to produce antigen-carrying bacterial OMVs in situ in the gut, exhibiting good therapeutic and preventive effects in pulmonary metastatic melanoma and subcutaneous colon tumour mouse models [206].

## The application of exosomes in clinical solid cancer immunity

Compared with stem cells, exosomes have the advantages of low toxicity, low immunogenicity, easy storage, high security, strong stability, no need to consider cellular activity, and penetration of the blood-brain barrier [212]. In recent years, exosome therapy has made great progress in the fields of regenerative medicine, oncology, drug transportation, immunotherapy, etc. A large number of studies have laid a solid foundation for its clinical application. At present, research on exosomes has entered the clinical trial stage (Table 1).

**Table 1:** Application of exosomes in clinical solid cancer immunity (Data source: ClinicalTrials.gov: https://beta.clinicaltrials.gov/ provided by the U.S. National Library of Medicine).

Cancer type	Application phase	Purpose of application	Clinical Trial No
Non-Small Cell Lung Cancer	Not Applicable	Prediction of immunotherapeutic effect of advanced NSCLC by detection of plasma exosomes	NCT04427475
Non-Small Cell Lung Cancer	Phase II (completed)	Trial of a vaccination with tumour antigen-loaded dendritic cell-derived exosomes	NCT01159288
Non-Small Cell Lung Cancer	Not Applicable	Exploring the consistency analysis of PD-L1 expression level detected in tissues and plasma exosomes (pExo), and guiding clinical practice of radiotherapy combining with immunotherapy	NCT02890849
Advanced Gastrointestinal Cancer	Unknown	Dynamic multiomics detection of plasma-derived exosomes to explore the efficacy and mechanism of anti-HER2, immunotherapy and anti-CLDN18.2 of gastrointestinal cancer.	NCT05427227
Advanced Gastric Carcinoma	Unknown	To verify the function of circulating exosomal lncRNA-GC1 on predicting and monitoring immunotherapeutic outcomes of GC	NCT05334849
Renal Cell Carcinoma	Unknown	To develop circulating exosomes as predictive biomarkers for the response to immunotherapy in RCC	NCT05705583
Hepatocellular Carcinoma	Unknown	Combined analysis of CTC and exosomes on predicting the efficacy of immunotherapy in patients with HCC	NCT05575622
Advanced HCC Metastatic Gastric Cancer Metastatic Colorectal Cancer	Phase I	CDK-004 is designed to allow for specific delivery of the STAT6 anti-sense oligonucleotide (ASO) to the myeloid to repolarize macrophage from immune suppressive M2 to proinflammatory M1 phenotype	NCT05375604
Advanced Solid Tumour	Phase I/II	A first-in-human, Phase 1/2 open-label, multicenter, dose escalation, safety, pharmacodynamic, and PK study of CDK-002 in subjects with advanced/metastatic, recurrent, injectable solid tumours	NCT04592484

With the deepening of research, exosomes have been found to play important roles in various health and disease models through molecular information transmission and are considered biomarkers and prognostic factors of diseases, with important clinical diagnostic significance for diseases. Exosomes related to PD-1/PD-L1 may be biomarkers for predicting the immunotherapy response. Research has found that the level of circulating exosomal PD-L1 was positively correlated with the level of IFN-γ and changed during anti-PD-1 therapy in melanoma patients. In the early stage of treatment, the increase in circulating exosome PD-L1 could serve as an indicator of the adaptive response of tumour cells to T-cell renewal, distinguishing between clinical responders and nonresponders [146]. The researchers carried out a prospective clinical trial to study whether the level of PD-L1 in plasma exosomes of melanoma patients can be used to predict patients’ response to immunotherapy. They found that the level of PD-L1 in circulating exosomes seemed to be a more reliable marker than the expression of PD-L1 in tumour biopsy. Tracking the change in PD-L1 in exosomes could monitor the disease progression of melanoma patients [213]. Studies have evaluated the expression of PD-1/PD-L1 on circulating exosomes in melanoma patients and found that higher levels of PD-1/PD-L1^+^ exosomes were significantly correlated with poorer progression-free survival (PFS) and overall survival (OS), which identified circulating PD-1^+^ exosomes as the driving factor of anti-PD-1 resistance and emphasized that analysing a single exosome population through liquid biopsy was a promising tool for immunotherapy stratification of melanoma patients [214]. Exosome biomarkers have been reported to be potential biomarkers for predicting the efficacy of anti-PD-1/PD-L1 immunotherapy in advanced NSCLC. Studies have explored the correlation between plasma exosomal miRNAs and prognosis in immunotherapy for EGFR/ALK-negative patients. The results indicated that the high expression of hsa-miR-320d, hsa-miR-320c, and hsa-miR-320b might be related to the adverse response of anti-PD-1/PD-L1 immunotherapy [215].

In addition to biomarkers for diagnosis and prognosis, exosomes also have broad prospects in the application of cancer treatment. Researchers loaded exosomes derived from autologous dendritic cells of malignant melanoma patients with melanoma-specific antigenic peptides to prepare tumour therapeutic vaccines and carried out phase I clinical trials to treat malignant melanoma, which confirmed the clinical safety of the vaccine [216]. Studies have shown that Dex could be applied in immunotherapy for NSCLC after chemotherapy [65]. Researchers conducted a phase II clinical trial on patients with advanced NSCLC who were unable to undergo surgical treatment. This phase II clinical trial confirmed that Dex enhanced the antitumour immune response of NK cells and improved the PFS of patients with advanced NSCLC [65]. In addition, researchers have used modified engineered exosomes to carry various types of bioactive molecules and target corresponding tumour tissues, activating the body’s own immune response to kill corresponding tumour cells, which have entered clinical trials. Researchers integrated stimulator of interferon genes (STING) agonists into the exosome and expressed PTGFRN protein on the exosome surface. The high level of PTGFRN expression could target the delivery of STING agonists to antigen-presenting cells in the TME, inducing the expression of interferon genes and locally activating the human immune response to kill tumour cells. The treatment of exoSTING (CDK-002) candidate drugs for patients with metastatic HNSCC, triple-negative breast cancer, anaplastic thyroid cancer and skin squamous cell carcinoma has carried out phase I/II clinical trials (clinicaltrials.gov NCT04592484). The researchers fixed a specific sequence of ASOs on the surface of exosomes and delivered it to highly immunosuppressive macrophages (M2 type) to reduce the expression of the immunosuppressive transcription factors STAT6 and C/EBP, thereby reprogramming M2-type macrophages into proinflammatory M1-type macrophages, activating macrophages and promoting an antitumour immune response. The treatment of exoASO-STAT6 (CDK-004) candidate drugs for patients with HCC, pancreatic ductal adenocarcinoma, colorectal cancer and other diseases has been carried out in phase I clinical trials (clinicaltrials.gov NCT05375604).

## Summary and outlook

In general, exosomes participate in the basic life processes of energy conversion, information recognition and transmission, and material transportation between different cells in life. The biological significance of exosomes has been deeply studied and has greatly developed in cancer diagnosis and treatment. Exosomes are closely related to the occurrence and development of tumours. Exosomes serve as diagnostic biomarkers, therapeutic targets, and drug delivery platforms and can be used for vaccine development.

In recent years, researchers have shown that exosomes can be detected and collected from blood, urine, saliva, and other body fluids. The enrichment of nucleic acids, proteins, lipids, and small-molecule metabolites in exosomes can reflect physiological and pathological changes, which provides a basis for exosomes as good biomarkers for non-invasive diagnosis and early screening of tumours. The ability of exosomes to penetrate the blood-brain barrier also provides a powerful tool for the early diagnosis of brain tumour patients. Moreover, the combination of exosomes and other liquid biopsy techniques is more helpful in improving the success rate of liquid biopsy and the accuracy of prediction.

Primary or secondary drug resistance is the main cause of tumour treatment failure. Therefore, there is an urgent need for biomarkers that can predict efficacy to screen the dominant treatment population and help the clinical quickly adjust the treatment strategy through real-time monitoring. Exosomes can mediate the resistance of almost all therapeutic drugs such as chemotherapy, targeting, and immunity. The changes in the expression levels of its contents are closely related to the treatment response, so it has great potential for therapeutic effect prediction and application prospects. Existing studies have shown that plasma exosomal PD-L1 not only affects the prognosis of patients but also is closely related to the response to immunotherapy. Therefore, the circulating exosomal PD-L1 is expected to become a new marker for predicting the efficacy of PD-1 inhibitor immunotherapy. Due to the fact that changes in blood biomarkers often occur earlier than imaging changes, achieving the clinical translation of exosome detection will have pivotal guiding significance for cancer treatment decision-making.

In addition to being liquid biopsy tools similar to CTC and ctDNA, exosomes are also highly anticipated research hotspots in immunotherapy for tumour as natural nanoscale vesicles. Exosomes have high biocompatibility and directional homing ability and can be modified according to target cells to increase directionality. Exosomes are more stable in body fluids than liposomes with similar structures and characteristics and can evade attacks from the immune system. Exosomes have a small volume and can penetrate biological barriers such as BBB. These characteristics determine that exosomes can serve as both targeted antitumour drugs and drug delivery carriers which are superior to conventional liposomes and artificial nanoparticles, assisting in drug delivery platforms and vaccine development for precise cancer treatments. In addition, inhibiting the biosynthesis and release of exosomes in tumour cells and reducing the uptake of exosomes by receptor cells may also block the information transmission mediated by exosomes, thus exerting antitumour effects. Therefore, exosomes are also considered to be potential targets for cancer treatment. The use of exosome inhibitors to inhibit the secretion of TDEs and weaken the immune suppression caused by exosomal PD-L1 provides an antitumour immunotherapy strategy targeting exosomes. Multiple studies have demonstrated that exosomes can simultaneously exert immune activation and immune suppression functions in tumours. Understanding their mechanisms can utilize their characteristics to facilitate cancer treatment. Due to the advantage of easily penetrating the surrounding tissues of tumour cells, NK-Exo and CAR-Exo have great potential in immunotherapy for solid cancers. Besides, Dex and antigen-modified TDEs may usher in a new era in the development of solid cancer vaccines.

Although exosome-based strategies have yielded benefits for improving the effects of anticancer immunotherapy, huge barriers remain in their clinical application for solid cancers. The main problem faced by the clinical transformation of exosomes is the cell source of exosomes and the preparation of industrialized exosomes. Unlike other chemical drugs, exosomes contain complex components. Currently, there is no technology that can extract exosomes with 100% purity. Therefore, the quality control of exosome products is also a major challenge at present. Shortening the time for separating and detecting exosomes and improving the purity of exosomes will help improve the efficiency of tumour liquid biopsy and precise tumour resection during surgery. In addition, achieving disease targeting by artificially modifying exosomes in solid tumours is also the key to improving the application value of exosome therapy products. Meanwhile, the combination of exosome inhibitors and ICIs provides a new direction for exosome-based antitumour immunotherapy. At present, the efficacy of exosome vaccines in immunotherapy for solid tumours is not satisfactory, possibly due to the T-cell dysfunction in TME, which leads to weaker immune responses. Therefore, seeking a certain combination therapy to enhance the activation effects of exosomes on T cells can help improve the immunotherapeutic effect of tumour patients. Although there are still some technical and functional bottlenecks that need to be addressed for exosomes as liquid biopsy tools and therapeutic drugs. In the future, exploring the pivotal role and unique characteristics of exosomes in the human body can boost the power of immunotherapy for solid cancers and offer one more step closer to clinical implementation. A large number of clinical trials and the development of drug delivery systems indicate that they will pave the way to overcome the challenges and dilemmas of immunotherapy for solid cancers.

### Supplementary information

Reproducibility checklist
